# Variation in serum biomarkers with sex and female hormonal status: implications for clinical tests

**DOI:** 10.1038/srep26947

**Published:** 2016-05-31

**Authors:** Jordan M. Ramsey, Jason D. Cooper, Brenda W. J. H. Penninx, Sabine Bahn

**Affiliations:** 1 Department of Chemical Engineering and Biotechnology, University of Cambridge, Cambridge, CB2 1QT, United Kingdom; 2 Department of Psychiatry, VU University Medical Centre and Neuroscience Campus Amsterdam, Amsterdam, The Netherlands; 3 Department of Neuroscience, Erasmus University Medical Centre, Rotterdam, The Netherlands

## Abstract

Few serum biomarker tests are implemented in clinical practice and recent reports raise concerns about poor reproducibility of biomarker studies. Here, we investigated the potential role of sex and female hormonal status in this widespread irreproducibility. We examined 171 serum proteins and small molecules measured in 1,676 participants from the Netherlands Study of Depression and Anxiety. Concentrations of 96 molecules varied with sex and 66 molecules varied between oral contraceptive pill users, postmenopausal females, and females in the follicular and luteal phases of the menstrual cycle (FDR-adjusted *p*-value <0.05). Simulations of biomarker studies yielded up to 40% false discoveries when patient and control groups were not matched for sex and up to 41% false discoveries when premenopausal females were not matched for oral contraceptive pill use. High accuracy (over 90%) classification tools were developed to label samples with sex and female hormonal status where this information was not collected.

Serum proteomic biomarkers have enormous potential for early diagnosis, quantification of disease risk, prognosis, and treatment response prediction and monitoring. They have been investigated for autoimmune disease and arthritis^1^,^2^, cancer^3^,^4^,^5^, cardiovascular disease^6^, mental and neurological disorders^7^,^8^,^9^,^10^,^11^,^12^, kidney function^13^, and other applications. Despite the increasing number of published serum biomarker studies (Supplementary Fig. 1), translation of these findings from the laboratory to clinical tools has proved challenging^14^,^15^. Only three novel biomarker tests on average are successfully validated and approved for clinical use by the US Food and Drug Administration (FDA) each year^16^. An important barrier to the clinical implementation of biomarker tests is their lack of reproducibility, the magnitude of which is of increasing concern^17^. In many cases this irreproducibility may be brought about by failing to properly consider variation in serum biomarkers caused by biological and lifestyle factors in study design and statistical analyses.

Although sex- and gender-based differences in medicine have gained attention in recent years, they are frequently overlooked in biomarker studies. A number of biological processes show substantial differences between males and females in various hormonal states, including endocrine^18^, nervous^19^, immune^20^, metabolic^21^, and cardiovascular^22^ function. Failing to account for sex and female hormonal status as important sources of variability in the concentrations of serum molecules may lead to confounding of these variables with disease status and reduce power to detect differences, contributing to the poor performance of biomarker tests in initial and follow-up studies.

In the present study, we investigated the effects of sex and female oral contraceptive pill (OC) use, phase of the menstrual cycle, and menopausal status on serum molecular abundance. We further evaluated the implications of not accounting for sex-based differences in biomarker studies by determining the potential number of false positive findings caused by confounding of disease status with sex or female use of OCs. Finally, we developed a classifier to label samples as males, OC users, postmenopausal females, or females with a menstrual cycle for retrospective analysis of biomarker studies where these variables were not initially recorded. We utilized serum samples from 1,676 well-characterized subjects (585 males and 1,091 females) from the Netherlands Study of Depression and Anxiety (NESDA)^23^. The concentrations of 171 proteins and small molecules measured using the Human DiscoveryMap® platform were examined, which is a pre-selected multiplex immunoassay panel that has been used for a number of biomarker studies (https://rbm.myriad.com; accessed October 2015). The panel was designed to investigate a broad range of analytes commonly associated with cancer, cardiovascular disease, kidney injury, neurodegenerative disorders, inflammation, and metabolic pathways. It comprises cytokines, acute phase reactants, hormones, growth factors, metabolic markers, tissue remodelling proteins, central nervous system markers, angiogenesis markers, and others. It will be important to incorporate this molecular information into future biomarker studies to improve the performance and reproducibility of these tests.

## Results

### Clinical samples: NESDA

We used serum samples collected from the Netherlands Study of Depression and Anxiety (NESDA) to investigate molecular concentrations in males and females taking the oral contraceptive pill (OC), in the follicular and luteal phases of the menstrual cycle, and after menopause. NESDA is a multi-site, naturalistic cohort study investigating the course of depressive and anxiety disorders in control and patient participants^23^. The Human DiscoveryMAP® multiplex immunoassay platform, a pre-selected panel of biomarkers relevant to disease, was used to measure the serum concentrations of 243 proteins and small molecules across 1,840 available NESDA participant samples. Of these, 164 samples were removed (see Methods) prior to analysis. Analyte assays with more than 30% missing values were removed, leaving 171 assays (see Supplementary Table 1 for a list of all 243 analytes and the percentage of missing values contained in each assay).

For the purposes of the study, we divided the data into a discovery cohort [unaffected controls; N = 347 (140 males/207 females)] and a validation cohort [patients with current and remitted anxiety and depressive disorders; N = 1,329 (445 males/884 females)]. We used a discovery cohort consisting of only unaffected controls in order to ensure that molecular sex and female hormonal status differences were not influenced by depressive/anxiety pathophysiology. Covariates recorded and used in later analysis included collection site, recruitment method, chronic disease, use of lipid modifying agents, use of anti-inflammatory drugs, use of antihypertensive medication, ancestry, education, physical activity, body mass index (BMI), age, alcohol consumption, smoking status, recreational drug use, partner status, and Inventory of Depressive Symptomatology (IDS) score (for more description of each see Supplementary Table 2). In the 347 NESDA discovery cohort samples, many of these demographic, lifestyle, and health variables differed between males, postmenopausal females, females using OCs, and females in the follicular and luteal phases of the menstrual cycle. They included age, BMI, waist circumference, alcohol consumption, presence of chronic disease, use of antihypertensive medication, blood pressure, IDS scores, collection area, and recruitment method (variables are summarized for males and females in Supplementary Table 3A). With the exception of IDS scores and collection area, these differences were also observed in the remaining 1,329 validation cohort subjects (Supplementary Table 3B).

### Molecular concentration variations with sex and female hormonal status

**Figure 1:**
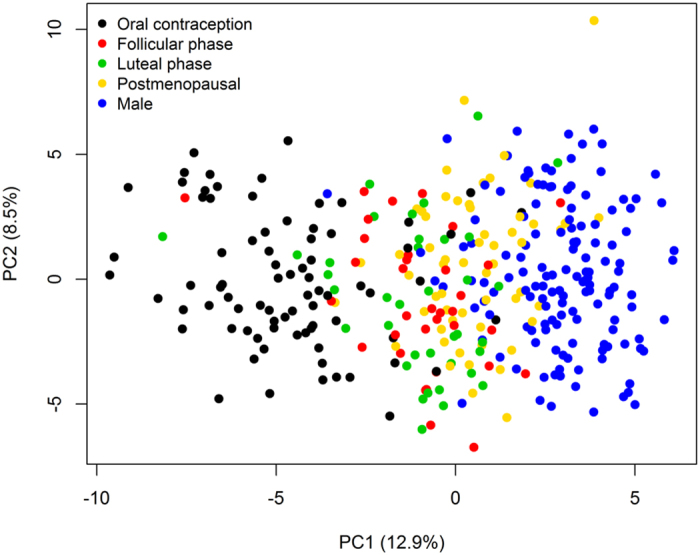
Principal component analysis (PCA) plot of NESDA discovery cohort samples. The first two principal components (PCs) are plotted and coloured according to sex and female hormonal status. PCA was performed using all analyte data. Percentage of variation accounted for by each principal component is shown in brackets with the axis label.

**Figure 2:**
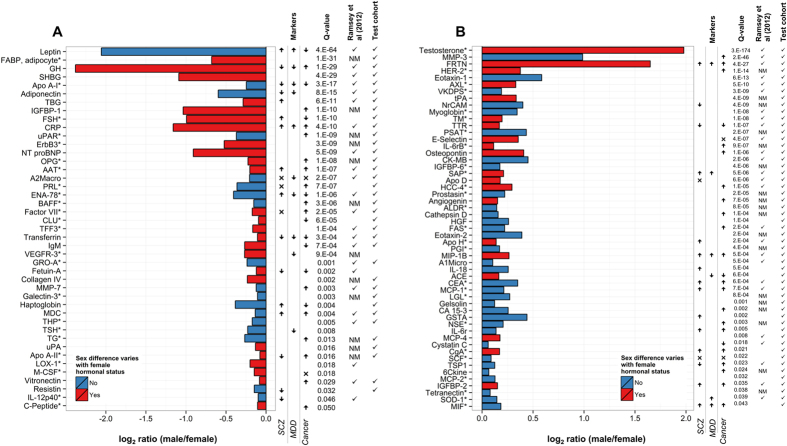
Serum analytes elevated significantly in (**A**) females and (**B**) males. Robust regression was used where *follows the analyte name. The log_2_ ratios of serum molecular concentrations in males compared to females shown in the plots were coefficients from the linear regression. Analytes in the plots are coloured in red where sex differences varied significantly with female hormonal status. Agreement with the validation cohort and our previous study is shown by a **✓** in the last columns. NM was used to indicate that the analyte was not measured in our previous study. To the right of the plots, biomarkers of schizophrenia (SCZ), major depressive disorder (MDD), and cancer are indicated by **↑** (elevated in patients); **↓** (reduced in patients); or × (conflicting evidence for elevated and reduced levels in patients). Analyte abbreviations can be found in Supplementary Table 1.

Principal component analysis (PCA) was conducted using analyte data from the discovery cohort (unaffected controls) and the first two principal components are plotted in Fig. 1. The plot illustrates the differences in serum molecular profiles between males and females for each hormonal status (classifications of female hormonal status included whether a female was in the follicular or luteal phase of the menstrual cycle, using OCs, or postmenopausal and not using hormone replacement therapy (HRT)). In the first principal component (accounting for 13% of variation), OC users and males showed the greatest separation between groups, with postmenopausal females and females in the follicular and luteal phases of the menstrual cycle located between these groups. The concentrations of 96 of the 171 investigated serum analytes differed between males and females in a linear model adjusting for covariates (listed above) that were selected using stepwise regression (Benjamini-Hochberg false discovery rate (FDR)-adjusted *p*-values (called *q*-values) less than 0.05 were considered significant). Of these, 43 had higher concentrations in females (Fig. 2A; Supplementary Table 4A) and 53 had higher concentrations in males (Fig. 2B; Supplementary Table 4B). Of these 96 molecular sex differences, 89 (93%) were also found in the 1,329 NESDA validation samples (Fig. 2; Supplementary Table 4). Findings from our previous study of molecular sex differences^24^ were also largely consistent with the current study. Out of the 60 assays measured in both studies, 49 findings (82%) were overlapping (Fig. 2; Supplementary Table 4).

**Figure 3:**
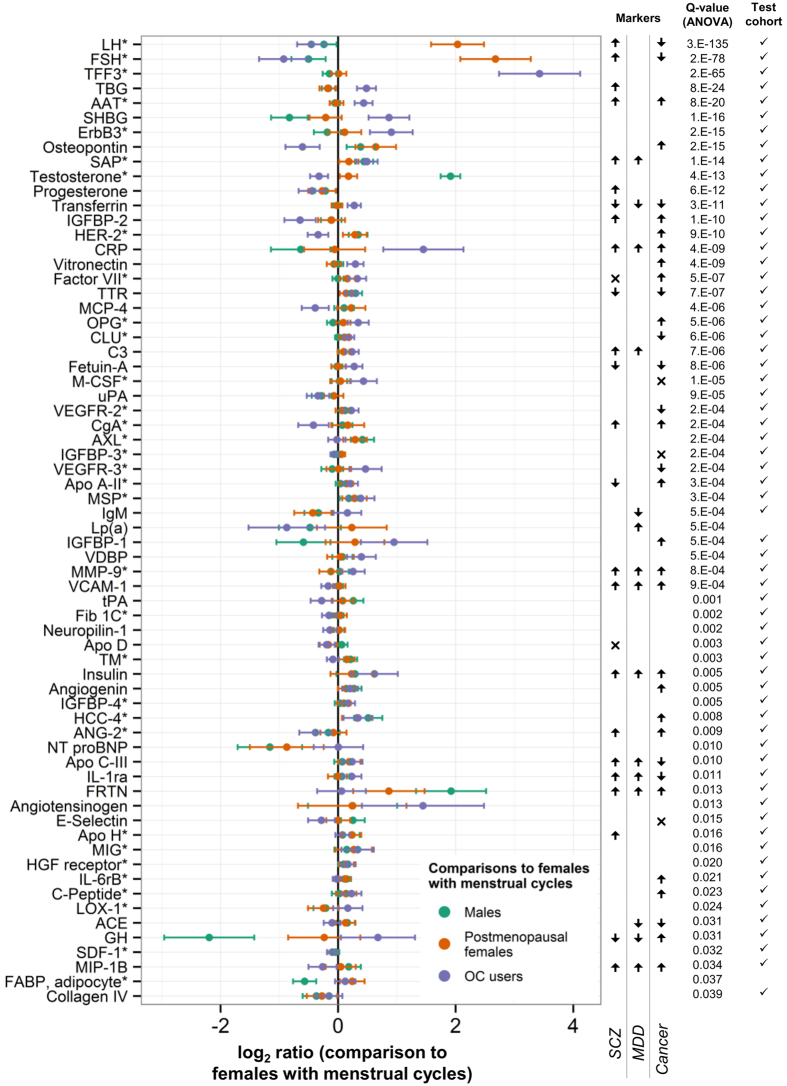
Serum analytes varying significantly with female hormonal status. Robust regression was used where *follows the analyte name. The log_2_ ratios of serum molecular concentrations of males, postmenopausal females, and females taking the oral contraceptive pill (OC) compared to females with a menstrual cycle, shown in the plot with their 95% confidence intervals, were coefficients from the linear regression. Agreement with the validation cohort is shown by a **✓** in the last column. To the right of the plot, biomarkers of schizophrenia (SCZ), major depressive disorder (MDD), and cancer are indicated by **↑** (elevated in patients); **↓** (reduced in patients); or × (conflicting evidence for elevated and reduced levels in patients). Analyte abbreviations can be found in Supplementary Table 1.

For 66 molecules, sex differences in serum concentrations varied significantly with female hormonal status (postmenopausal, OC use, follicular and luteal phases of the menstrual cycle; Fig. 3; Supplementary Table 5). Sixty-four (97%) of these were replicated in the NESDA validation cohort, consisting of 1,329 samples (Fig. 3; Supplementary Table 5). The levels of 55 of the 66 serum analytes differed between OC users and females with menstrual cycles, and 26 molecules differed between postmenopausal females not using HRT and females with menstrual cycles. Figure 3; Supplementary Table 5 shows that for most of the significant differences between postmenopausal females and females with menstrual cycles, postmenopausal analyte levels tended toward male levels. Only five of the 66 serum analytes differed significantly between females in the luteal and follicular phases of the menstrual cycle (Supplementary Table 5). Follicle-stimulating hormone (FSH), neuropilin-1, and insulin-like growth factor binding proteins (IGFBP)-2 and -3 were increased in the follicular phase, while progesterone was increased in the luteal phase. A total of 117 of the 171 (68%) analyzed analytes showed significant sex differences and/or varied with female hormonal status.

### Potential relevance to biomarkers of disease

The 117 analytes that varied significantly with sex and female hormonal status were involved in a number of important biological processes, including metabolic processes, developmental processes, cell communication and signal transduction, transport, growth and cell proliferation, chemotaxis, response to stress, cell death, defence, inflammatory, and immune response, nervous system development, vasculature development, and others (determined from gene ontology (GO) biological process (BP) terms; see Supplementary Table 4 and Supplementary Table 5). The relevance of our findings to potential biomarkers of disease was also illustrated by investigating their relationships to proposed biomarkers of mental disorders and of cancer. Lists of reported plasma or serum markers of schizophrenia^8^,^9^,^10^,^11^,^12^, MDD^7^,^8^, and various cancers^3^,^4^,^5^ were compiled. In total, of the 117 analytes that differed between males and females or with female hormonal status, 45 were previously reported to be changed in schizophrenia patients, 23 were changed in MDD patients, and 60 were changed in cancer patients (Figs 2 and 3).

**Figure 4:**
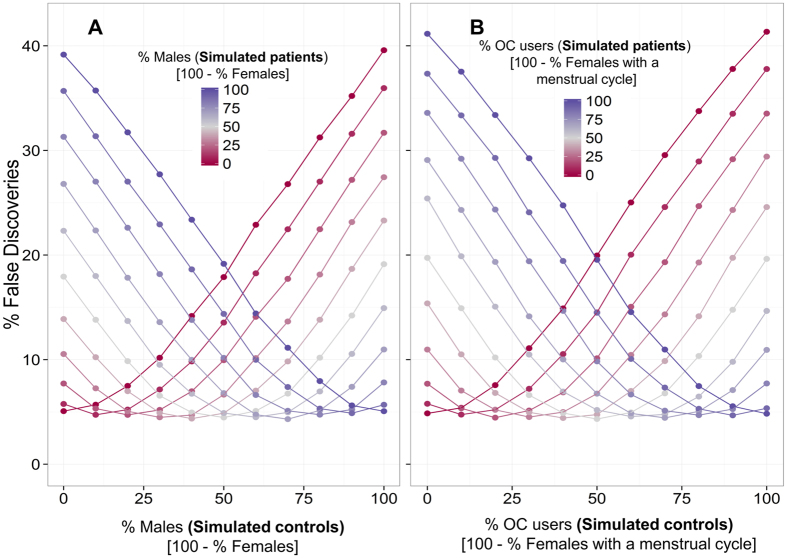
Average percentage of analytes falsely discovered between simulated patient and control groups with varying proportions of (**A**) males compared to females and (**B**) oral contraceptive pill (OC) users compared to females with menstrual cycles. Simulations represented biomarker studies for which there were no serum molecular differences between patient and control groups. For (**A**,**B**), the composition of the control group is shown on the x-axis, while the composition of the patient group is shown by lines of varying colours. Blue lines represent simulations with more (**A**) males or (**B**) OC users in the patient group, while red lines represent simulations with more (**A**) females or (**B**) females with menstrual cycles in the patient group. The y-axis shows the average percentage of analytes falsely discovered (*p* *<* 0.05) in each simulation.

Simulations were conducted to represent biomarker studies for which disease/control status had no effect on serum molecular levels, but which yielded false positive results due to confounding with sex or female hormonal status. These simulations showed that when groups were not matched for sex, up to 39.6% of measured analytes were falsely discovered, as seen in Fig. 4A. Furthermore, when the proportion of OC users differed between patient and control groups of premenopausal females, up to 41.4% of measured analytes were false discoveries (Fig. 4B). Less severe confounding also had the potential to produce a substantial fraction of false positive results. For example, comparing a group of premenopausal female control subjects consisting of 20% OC users to a group of patients consisting of 60% OC users caused false discoveries in 15% of serum molecules on average. For comparison, in balanced groups there was a false discovery rate of 4.6% of analytes on average.

### Classification of sex and female hormonal status

**Figure 5:**
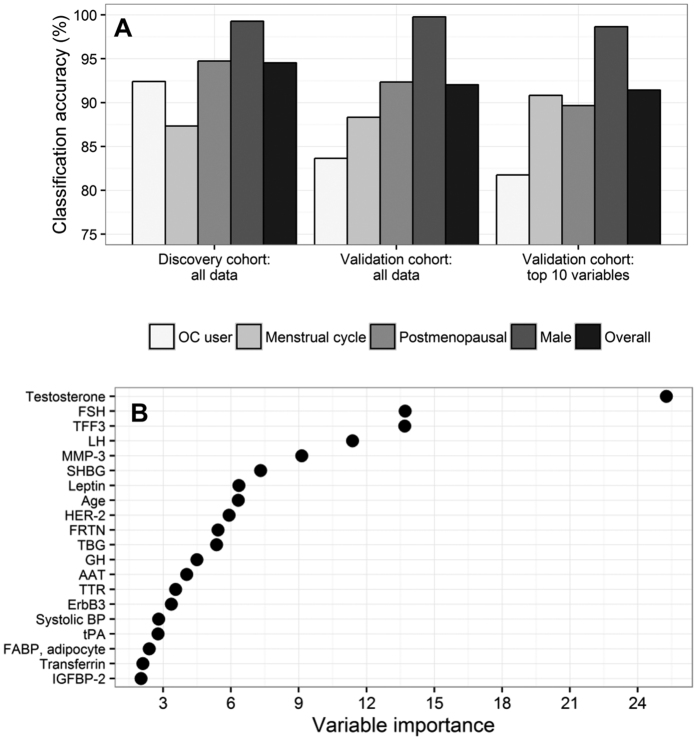
Classification accuracy (**A**) and variable importance (**B**) for the random forest classifiers constructed to predict sex and female hormonal status using serum analyte measurements and other covariates in the discovery cohort. Female hormonal status included oral contraceptive pill (OC) users, females with a menstrual cycle, and postmenopausal females. The two leftmost plots in (**A**) show accuracies for the random forest classifier constructed using all data (189 predictors), while the rightmost plot shows accuracies for the random forest classifier constructed using only the ten most important variables for classification, which are shown in (**B**). Variable importance was assessed by the mean decrease in the Gini impurity criterion (a measure of node purity; more details in the Supplementary Methods). Abbreviations: BP (blood pressure). Analyte abbreviations can be found in Supplementary Table 1.

We next used random forests to construct classifiers to label samples as male, postmenopausal female, OC user, or female with a menstrual cycle using covariate and analyte data from the discovery cohort. For the first classifier constructed using all 171 analytes and 18 covariates, classification accuracies of 94.5% were achieved for the discovery cohort data and 92.0% for the 1,329 test samples from the validation cohort (Fig. 5A; Supplementary Table 6). Classification accuracies were above 80% for all groups in both discovery and validation cohorts, but were higher for males and postmenopausal females than for OC users or females with a menstrual cycle. Testosterone, FSH, trefoil factor 3 (TFF3), luteinizing hormone (LH), matrix metalloproteinase-3 (MMP-3), sex hormone-binding globulin (SHBG), leptin, age, human epidermal growth factor receptor 2 (HER-2), and ferritin were the ten most important variables for classification of sex and female hormonal status, using the full set of analytes and covariates (Fig. 5B). These ten variables were used to construct the second random forest classifier. Similar classification accuracies were achieved for this reduced classifier (91.4% overall accuracy in the validation cohort; Fig. 5A and Supplementary Table 6C) compared to the first classifier constructed from the full set of 189 predictors.

## Discussion

This investigation represents a substantial contribution to our understanding of sex and female hormonal status (including use of the oral contraceptive pill (OC), menopause, and menstrual cycle phase) as sources of variation in serum molecular concentrations and their considerable impact on biomarker studies.

Principal component analysis first showed that sex and female hormonal status contributed most to the variability in analyte levels. In total, we found that the serum concentrations of 117 out of 171 (68%) molecules were associated with sex and/or female hormonal status, adjusting for variables such as age, BMI, medication use, and other relevant demographic, lifestyle, and health variables. Molecules were involved in endocrine, immune, metabolic, growth, and other processes. These findings may emerge as a direct result of sex chromosome effects and indirectly through levels of sex hormones^25^. Organization and activation effects of sex steroids act through genomic and non-genomic mechanisms^26^ to affect a broad range of biological processes, including brain development and function^19^, immune response^20^, cardiovascular function^22^, other endocrine systems^18^, metabolism of glucose, lipids^21^, and drugs^27^, and others. The results found here were highly reproducible, with 94% of all findings replicated in a validation cohort and 89% of molecular sex differences consistent with our previously published study^24^. The importance of these findings is illustrated by the fact that many of the identified molecules are published biomarkers for cancer, schizophrenia, and major depressive disorder (MDD). Apart from a potential utility for biomarker tests, these changes provide insights into disease-specific sex differences including a higher overall risk of cancer in males^28^, a higher prevalence of MDD in females^29^, a higher incidence of schizophrenia in males with a later female age of onset^30^, and a wide range of disorders with sex differences in prevalence, symptoms, and response to treatment. For example, the higher overall risk of cancer in males may be associated with their increased levels of human epidermal growth factor receptor (HER)-2, AXL receptor tyrosine kinase^31^, stem cell factor (SCF), matrix metalloproteinase-3 (MMP-3)^32^, thrombospondin 1 (TSP1), and/or angiogenin^33^ found in this study.

Almost half of the molecules found to have significantly different concentrations between males and females also varied depending on the hormonal status of females. In particular, extensive differences in serum molecular concentrations of oral contraceptive pill (OC) users were identified compared to females with menstrual cycles. These findings may be associated with the reduced all-cause mortality^34^, lower risk of endometrial and ovarian cancer and bacterial pelvic inflammation^35^, increased risk of breast^36^, cervical^37^, and liver^38^ cancers, higher risk of certain cardiovascular events^35^, increased incidence of Crohn’s disease and ulcerative colitis^39^, and potential effects on symptoms of depression^40^ in OC users. Increased levels of the inflammatory biomarker C-reactive protein (CRP) found in OC users in this study may contribute to their higher risk of cardiovascular events^41^ and higher CRP and TFF3 in OC users may be linked to their increased risk for Crohn’s disease/ulcerative colitis^42^,^43^,^44^. Furthermore, the higher serum level of HER-3/ErbB3^45^, lower level of HER-2^46^, and changes in the levels of IGFBPs^47^ and vascular endothelial growth factor receptors (VEGFRs)^48^ in OC users may provide insight into their differential risks for certain cancers.

Changes in molecular serum concentrations in postmenopausal females not using HRT largely tended towards the levels found in males. Among others, these included thyroxine binding globulin, transthyretin, AXL receptor tyrosine kinase, ferritin, serum amyloid component P-component, HER-2, osteopontin, and immunoglobulin M. These could play a role in the increased prevalence of many conditions after menopause, including cardiovascular disease^49^, osteoporosis^50^, metabolic syndrome^51^, Alzheimer’s disease, and late-onset schizophrenia^52^. On the other hand, few differences were observed between females in the follicular and luteal phases of their menstrual cycles. As expected, we found that FSH was elevated in the follicular phase and progesterone was elevated in the luteal phase of the menstrual cycle. However, several factors, including individual differences in the timing and length of phases, smaller numbers of females in these groups, unreliable self-reporting of menstrual cycle status, and complex variability in hormonal levels within phases likely contributed to the lack of findings. In particular, the menstrual cycle phase classification used here could not account for variation in hormone levels prior to ovulation, in which peak levels of estrogen, LH, and FSH are attained^53^. These results should therefore be interpreted with caution as they likely do not adequately represent the full extent of the effects of menstrual cycle variation.

Simulations performed in this study support the practice of designing studies to match cases and controls for sex, with the most severe discrepancy in the proportion of males and females between simulated patient and control groups yielding almost 40% false positive results. Although many biomarker studies already match for sex, most do not match premenopausal females for OC use, despite being used by a substantial proportion of females in the developed world. Our simulations showed that this omission represents an important potential source of false discovery. Up to 41% false positive results were obtained when groups of premenopausal females were not matched for OC use. A PubMed search (*August 2015*) revealed that none of the twenty most recent published cancer serum biomarker studies reported use of OCs or other female hormonal status information for patients or controls. Recent serum proteomic biomarker studies of hepatocellular carcinoma^54^, pancreatic cancer^55^, ovarian cancer^56^, breast cancer^57^, and gastric cancer^58^ reported sex of clinical samples, but not OC use or other measures of hormonal status in females. Imbalances in OC use between patient and control groups may be more likely when investigating cancers for which use of OCs increases or decreases risk, such as ovarian or breast cancer, and in mental disorders for which contraceptive use is less consistent^59^.

Apart from reducing the number of false discoveries in biomarker studies, accounting for sex and female hormonal status can also increase the power of analyses by accounting for this source of variability in analyte concentrations. Furthermore, personalized clinical biomarker cut-off points could improve classification accuracy of conditions for these groups. For example, we found that high CRP is a biomarker of schizophrenia, MDD, and cancer. However, higher serum concentrations were measured in females, and particularly in OC users. Use of a common CRP cut-off point for classification may result in a greater rate of false positives in females and false negatives in males.

In light of these considerations, high accuracy classification tools were developed to assist in labelling samples as males, postmenopausal females, OC users, and females with a menstrual cycle where this information has not been collected. Classification using age and the concentrations of nine serum analytes was effective in labelling the validation cohort with an overall accuracy of 91%. Future studies should be designed to collect this information, but our classifier may provide a means of re-evaluating past biomarker studies accounting for sex and female hormonal status where this was not initially recorded.

Certain limitations of this study should be taken into account. Estrogen dose, progestogen type, brand, and monophasic or triphasic OC use were not recorded. We note that Dutch medical guidelines recommend that if a combined OC is chosen, preference is for one with levonorgestrel and 30 μg of ethynyl estradiol^60^. Active-pill or hormone-free interval for OC users, duration of OC use, and timing of menopause were also not recorded and should be considered in future studies. The crude classification of menstrual cycle phase used here was a key limitation to understanding the effects of menstrual cycle variation. Alternative models capturing more points across the cycle should be evaluated in future. Pregnant and breastfeeding females, females who had undergone a hysterectomy, and individuals undergoing treatment with sex hormones other than OCs were not evaluated in this investigation. These subjects comprised only approximately 5% of the samples in this study, but may be more important for investigating certain populations. Finally, NESDA is an observational study specifically designed to investigate depressive and anxiety disorders. This necessitated division of the data into discovery (unaffected control) and validation (patient) groups to ensure that molecular sex differences were not influenced by depressive/anxiety pathophysiology, reducing power to detect differences.

This study provides a valuable resource to investigators wishing to improve or produce robust serum biomarker tests for diagnosis, monitoring, and prediction of disease and response to treatment. Researchers examining specific proteins should draw on this and other resources^61^ in order to design studies incorporating all known factors shown to affect their particular biomarkers of interest. We show that accounting for both sex and female hormonal status in the design and statistical analysis of biomarker studies is an essential step towards obtaining reproducible results, establishing appropriate clinical cut-offs for men and women, and improving the translation of much needed biomarker tests to the clinic. We believe this investigation also serves a complementary purpose of informing researchers investigating differences in the risks of certain diseases and disorders between males and females across hormonal states. The sex differences evaluated here provide a foundation for further biomarker research aimed at improving the diagnosis and treatment of men and women.

## Methods

### Clinical samples: NESDA

The Netherlands Study of Depression and Anxiety (NESDA) includes 2,981 control and patient participants aged 18–65 years, recruited from the community (19%), general practice (54%), and secondary mental health care (27%) between 2004–2007^23^. The study protocol for NESDA was carried out in accordance with guidelines approved by the Ethical Review Board of the VU University Medical Centre and by local review boards at each participating centre. Informed written consent was given by all participants.

In women, use of oral contraceptive pills (OCs) and sex hormones was assessed by self-report in NESDA. OCs were combined (estrogen and progestogen) or progestogen only with varying doses of estrogen, different types of progestogen, and were monophasic or triphasic. Classification of menstrual cycle phase in women [follicular (0–13 days of the cycle) and luteal (14–32 days or more)] and status as postmenopausal, hysterectomized, or pregnant/breastfeeding were also self-reported. It should be noted that the crude classification of menstrual cycle phase used here did not account for complex hormonal variations across the cycle, individual differences in timing and length of phases, or unreliable self-reporting of cycle day. Women who were pregnant or breastfeeding, using sex hormones (ATC code G03) not classified as OCs, had undergone a hysterectomy, or with missing information on hormonal status were excluded from further analysis. These and other demographic, lifestyle, and health variables used in subsequent analyses are listed below and further described in Supplementary Table 2. A summary of these variables for males and females in the follicular and luteal phases of the menstrual cycle, using OCs, and after menopause is reported in Supplementary Table 3.

Diagnoses of lifetime and current depressive disorders (major depressive disorder (MDD) and dysthymia) and anxiety disorders (social phobia, generalized anxiety disorder, panic disorder, and agoraphobia) were carried out during a baseline interview by specially trained research staff using the Composite Interview Diagnostic Instrument (CIDI, World Health Organization (WHO) version 2.1) in accordance with Diagnostic and Statistical Manual of Mental Disorders (DSM)-IV criteria. Control subjects (discovery cohort) had neither a current nor a lifetime diagnosis of the psychiatric disorders evaluated and did not develop any assessed disorder by the second year follow-up assessment. The remaining patients were used for validation (as stated above). Subjects were excluded from NESDA when not fluent in the Dutch language and when they had a primary clinical diagnosis of other psychiatric disorders not studied in NESDA: bipolar disorder, obsessive compulsive disorder, severe substance use disorder, or psychotic disorder. Further details of the NESDA study design, protocol, and assessed participant information have been previously described^23^.

### Multiplex immunoassays

Blood samples from NESDA were collected at the baseline assessment in the morning at approximately 0800 hours after an overnight fast. Serum samples were stored at −80 °C until analysis. The Human DiscoveryMAP® multiplex immunoassay platform was used to measure the serum concentrations of a total of 243 analytes in 1,840 NESDA participant samples assigned to 26 plates. Remaining samples were excluded based on blood sample quality (N = 756) and lack of follow-up data (N = 385; see also Supplementary Figure 2). A stratified randomization of samples to plates was carried out to ensure disorders were approximately evenly distributed across them. The established multiplex immunoassay panel contained biomarkers relevant to cancer and other diseases and included cytokines, hormones, growth, factors, metabolic markers, and more (see Supplementary Table 1). Protocol for this procedure is described in greater detail elsewhere^62^. Of the 1,840 samples measured, three were removed after internal quality control checks. Average intra-assay variability was 5.6% (2.5–15.8%) and inter-assay variability was 10.6% (5.5–32.5%)^62^.

### Data pre-processing

Data pre-processing and analysis was carried out using R (v3.1.2)^63^. Analyte assays with more than 30% missing values in the 1,837 samples were first removed. This resulted in exclusion of 72 assays from the panel of 243, leaving 171 for further analysis. A list of the 243 analytes and the percentage of missing values for each assay is found in Supplementary Table 1. One sample was removed from the study with more than 30% missing assays. Missing values for the remaining assays were replaced by the minimum or maximum analyte level for measurements below or above the limit of quantitation, respectively. Analyte values that were missing due to low sample volume were replaced by the mean concentration for that analyte. We replaced missing covariate data (physical activity: 5.0% missing, alcohol consumption: 0.8% missing, Inventory of Depressive Symptomatology (IDS) score: 0.7% missing, and recreational drug use: 0.5% missing) with the mean or most frequent value for continuous and discrete variables, respectively.

To adjust for batch effects caused by running samples on different plates, we used ComBat after log_2_ transforming the analyte data, implemented in the sva package in R^64^. ComBat is an empirical Bayes method of adjusting for additive and multiplicative batch effects and has been used in microarray data^65^. Multivariate outliers were assessed based on a robust measure of the Mahalanobis distance, calculated using the robust package in R^66^, resulting in the removal of four samples.

### Sample exclusion

After data pre-processing, 1,832 samples remained for analysis. Additional samples were excluded for the following reasons: 1) the participants had not fasted when blood was withdrawn (N = 56); or the participants were females who 2) were pregnant or breastfeeding (N = 10), 3) used sex hormones (ATC code G03) not classified as OCs (N = 18), 4) had undergone a hysterectomy (N = 61), and 5) had missing hormonal status information (N = 11). This left 1,676 samples for further analysis, consisting of 347 control subjects in the discovery cohort (140 males/207 females) and 1,329 patients (445 males/884 females) in the validation cohort. Supplementary Figure 2 illustrates sample exclusions carried out at each stage of analysis.

### Data analysis

The first two principal components (PCs) from a principal component analysis (PCA) of all analyte data from the discovery cohort were first used to visualize patterns of variation arising with sex and female hormonal status (here, classifications of female hormonal status were whether a female was in the follicular or luteal phase of the menstrual cycle, using OCs, or postmenopausal and not using HRT). The first two PCs, accounting for the highest proportions of variation, were used to simplify plotting, visualization, and interpretation of the PCA. PCA was performed using the FactoMineR package in R^67^. A linear model was then used to evaluate sex differences in the concentrations of analytes in the discovery cohort (N = 347). The analysis considered collection site, recruitment method, chronic disease, use of lipid modifying agents, use of anti-inflammatory drugs, use of antihypertensive medication, ancestry, education, physical activity, body mass index (BMI), age, alcohol consumption, smoking status, recreational drug use, partner status, and IDS score as potential additional covariates (see Supplementary Table 2 for more information and Supplementary Table 3 for a summary of demographic and other information for males and females). Covariates were selected using stepwise regression with simultaneous forward selection and backward elimination using Bayesian Information Criterion (BIC). Robust regression (MM-estimator) was used where regression outliers were present (with Bonferroni-corrected *p*-value < 0.05), performed using the robustbase package in R^68^.

In order to investigate variation in the concentration of serum analytes with female hormonal status, analysis of variance (ANOVA) was used to evaluate the effect of adding these classifications to the linear model. Females were classified as being in the follicular (N = 34) or luteal (N = 37) phase of the menstrual cycle, an OC user (N = 79), or a postmenopausal female not using HRT (N = 57). Where robust regression was used, ANOVA used a robust Wald-type test implemented in the robustbase package in R^68^. When the ANOVA was significant, contrasts were then used to compare the levels of serum analytes in: 1) females using OCs compared to females with a menstrual cycle (females in the follicular and luteal phases of menstruation), 2) postmenopausal females compared to females with a menstrual cycle, 3) males compared to females with a menstrual cycle, and 4) females in the follicular compared to the luteal phase of the menstrual cycle.

We then applied the same analyses to the 1,329 subjects in the validation cohort, which contained 445 males, 263 OC users, 149 females in the follicular phase and 211 in the luteal phase of the menstrual cycle, and 261 postmenopausal females not using HRT. Findings from the discovery cohort were compared to validation cohort results and to the results of our previous study of serum molecular sex differences^24^. Previously, we found sex differences in the levels of 77 serum molecules in 392 subjects (196 males and 196 females) free of chronic diseases. It should be noted that demographic and lifestyle information was limited and hormonal status of females was unavailable in this study. Adjusted *p*-values were calculated for each analysis to account for multiple testing using the Benjamini and Hochberg false discovery rate (FDR) procedure^69^. These FDR-adjusted *p*-values are reported as *q*-values. *Q*-values were considered significant at the 5% level.

The Gene Ontology (GO) Consortium provides a dynamic, controlled vocabulary for annotating genes based on three independent ontologies: cellular components, molecular functions, and biological processes (BP)^70^. Biological processes for significant proteins were investigated using GO BP terms. These were found using the R Bioconductor package Uniprot.ws^71^ and mapped to all ancestor terms using the *GOSim* package^72^.

### Simulation of group imbalance of sex and female hormonal status

In order to assess the effect of ignoring sex and female hormonal status on false discoveries in biomarker studies, simulations were performed. These simulations imitated biomarker studies of disease and control subjects for which there were no differences in serum molecular concentrations. Patient status was confounded with sex or female hormonal status in our simulations, therefore potentially giving rising to false discoveries of disease biomarkers. In the first set of simulations, we modelled imbalances in the number of males and females in disease and control groups. For both groups, the sex composition was varied for each simulation by 10%, with group compositions ranging from 0% to 100% males. For each simulation, 50 samples each were selected with replacement from male (N = 140) and female (N = 207) discovery cohort subjects to represent the disease and control groups. A *t*-test was used to test for differences between the two groups in each of the 171 analyte levels and the number of false discoveries (*p*-value < 0.05) was recorded. One thousand simulations were performed for each male/female group composition and the average percentage of false discoveries was calculated. In the second simulation, this procedure was repeated to investigate potential false discoveries in disease and control groups of females with an imbalance in the composition of reproductive-aged OC users.

### Classification of sex and female hormonal status

Data from the 347 discovery cohort subjects were used to build two random forest classifiers to distinguish between males, postmenopausal females, OC users, and females with a menstrual cycle. For the first classifier, all 171 serum analytes were used as predictors together with all covariates used in the linear models, apart from recruitment method. Waist circumference and systolic and diastolic blood pressure were also used, totalling 189 predictors (171 analytes and 18 covariates) for the first classifier. Five thousand trees were constructed using the randomForest package in R^73^ and the importance of each variable for classification was assessed with the mean decrease in the Gini impurity criterion, a measure of node purity (more details in the Supplementary Methods). The out-of-bag (OOB) error, an estimate of the error on unseen data, was assessed (more details in the Supplementary Methods). The remaining 1,329 NESDA samples in the validation cohort were also used to test the performance of this classifier. The second classifier was constructed using only the ten most important variables from this first classification, again using the randomForest package with 5,000 trees. Its performance was tested in the validation cohort (consisting of 1,329 NESDA samples).

## Additional Information

**How to cite this article**: Ramsey, J. M. *et al.* Variation in serum biomarkers with sex and female hormonal status: implications for clinical tests. *Sci. Rep.* **6**, 26947; doi: 10.1038/srep26947 (2016).

## Supplementary Material

Supplementary Information
