# Supplementary material for: Variation in serum biomarkers with sex and female hormonal status: implications for clinical tests
Source: Sci Rep. 2016 May 31;6:26947. doi: 10.1038/srep26947 (PMC4886262; doi:10.1038/srep26947)
Supplement: Supplementary Information [file srep26947-s1.pdf]

Supplementary Information

Variation in serum biomarkers with sex and female hormonal status: implications for clinical tests

Jordan M Ramsey[a], Jason D Cooper[a], Brenda WJH Penninx[b*], Sabine Bahn[a,c*]

[a]Department of Chemical Engineering and Biotechnology, University of Cambridge, Cambridge, CB2 1QT, United Kingdom

[b]Department of Psychiatry, VU University Medical Centre and Neuroscience Campus Amsterdam, Amsterdam, The Netherlands

[c]Department of Neuroscience, Erasmus University Medical Centre, Rotterdam, The Netherlands

*Shared last authors

Supplementary Methods

Further description of random forests

Random forests were used in this study to classify observations as males, postmenopausal females, females taking the oral contraceptive pill (OC), or females with a menstrual cycle using analyte concentrations and other demographic variables. This ensemble method constructs many classification trees and classifies new observations based on the majority decision (majority vote) of these trees[1]. Each tree in the forest is grown from n random cases sampled with replacement (from n observations) by recursively splitting observations until the terminal node contains data purely from one category[2,3]. The maximum decrease in the Gini impurity criterion, a measure of node homogeneity, determines the best way to split the data at each node from $\sqrt{p}$ (of p total) randomly selected variables[2,3]. The Gini impurity criterion is[4]:

$$I_G = 1 - \sum_{i=1}^{m} f_i^2$$

where f_i is the fraction of observations with label i in the set, of a total of m possible labels. Greater node homogeneity occurs with smaller values of I_G, which reaches zero when the node contains observations from only one category. Out-of-bag (OOB) error gives an unbiased estimate of classification error in unseen (test) samples in random forests. It is calculated by classifying each sample using those trees from the random forest not constructed with that sample. **Figure 1** shows a simulated example of a single decision tree for classifying an observation as either disease or control based on $\log_2$-transformed analyte concentrations. Random forests were composed of 5000 trees and built using the randomForest function in the R package of the same name[3]. Variable importance was assessed by adding the decreases in Gini impurity resulting from a split on a particular variable and averaging over the number of trees[2].

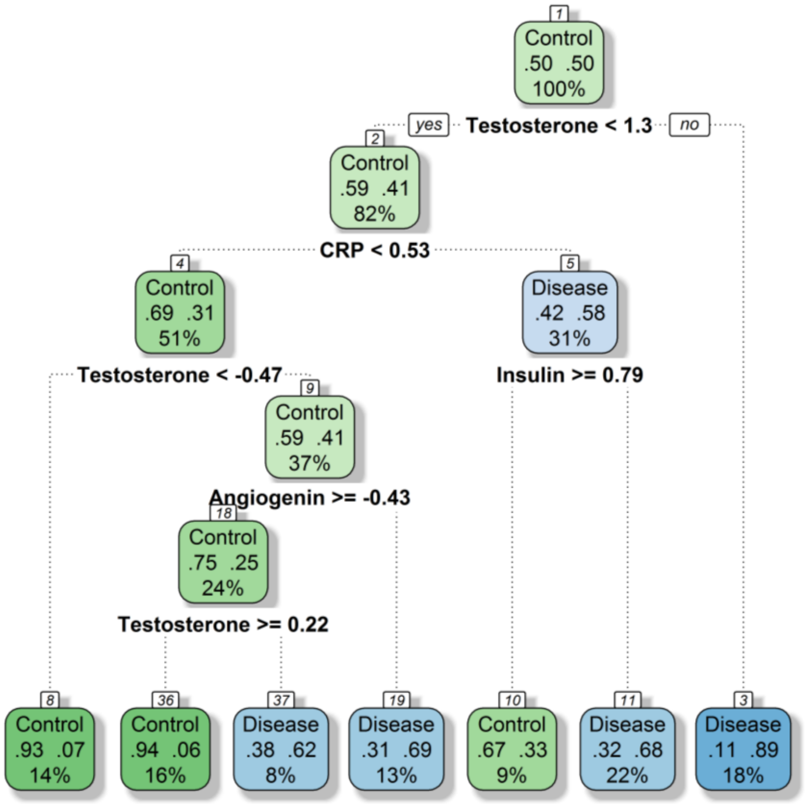

Figure 1. **Simulated decision tree for classifying an observation as either disease or control based on $\log_2$-transformed analyte concentrations.** For each node, the top line shows the enriched class; the middle line shows the proportion of each class; and the bottom line shows the percentage of observations. The decision boundary is shown below each node. Only part of the decision tree is shown (terminal nodes are not shown).

References

1. Breiman, L. Random Forests. *Mach. Learn.* **45,** 5–32 (2001).

2. Breiman, L. *Manual on Setting Up, Using, and Understanding Random Forests V3.1*. (2002).

3. Liaw, A. & Wiener, M. Classification and Regression by randomForest. *R News* **2,** 18–22 (2002).

4. Hastie, T., Tibshirani, R. & Friedman, J. *The Elements of Statistical Learning: Data Mining, Inference, and Prediction*. (Springer, 2009).

Supplementary Figures

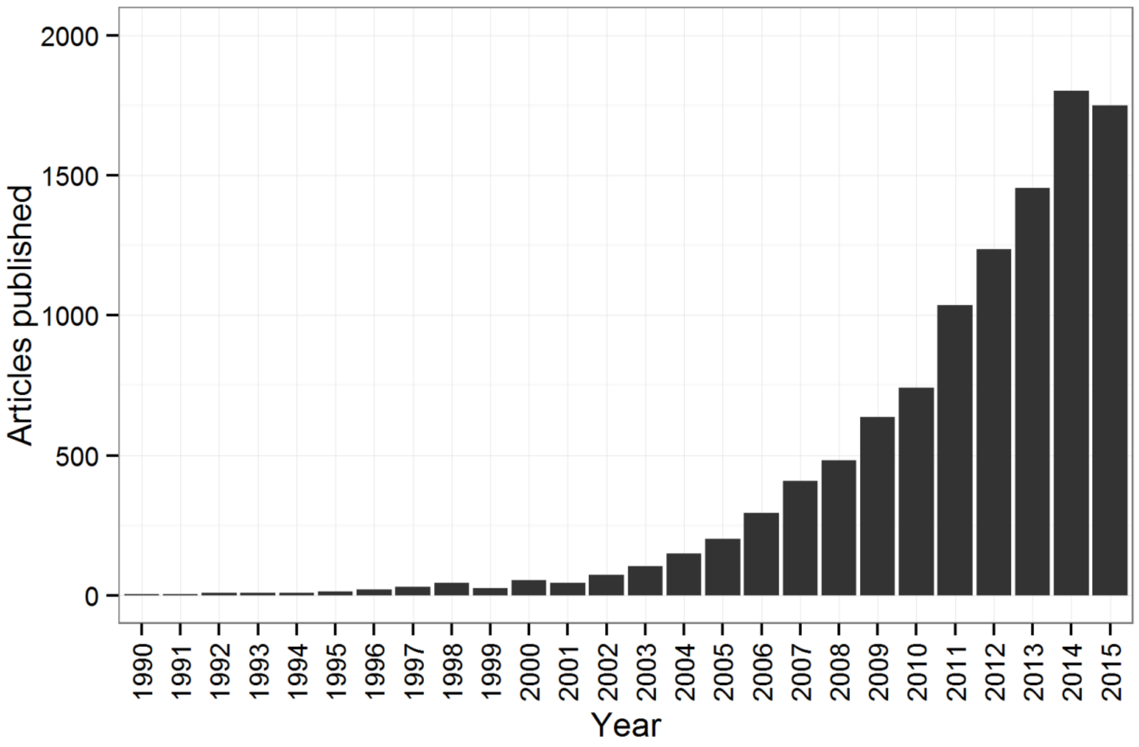

Supplementary Figure 1. Number of articles published each year containing the terms "biomarker" and "serum" in the title or abstract. Publications were searched in September 2015 using PubMed.

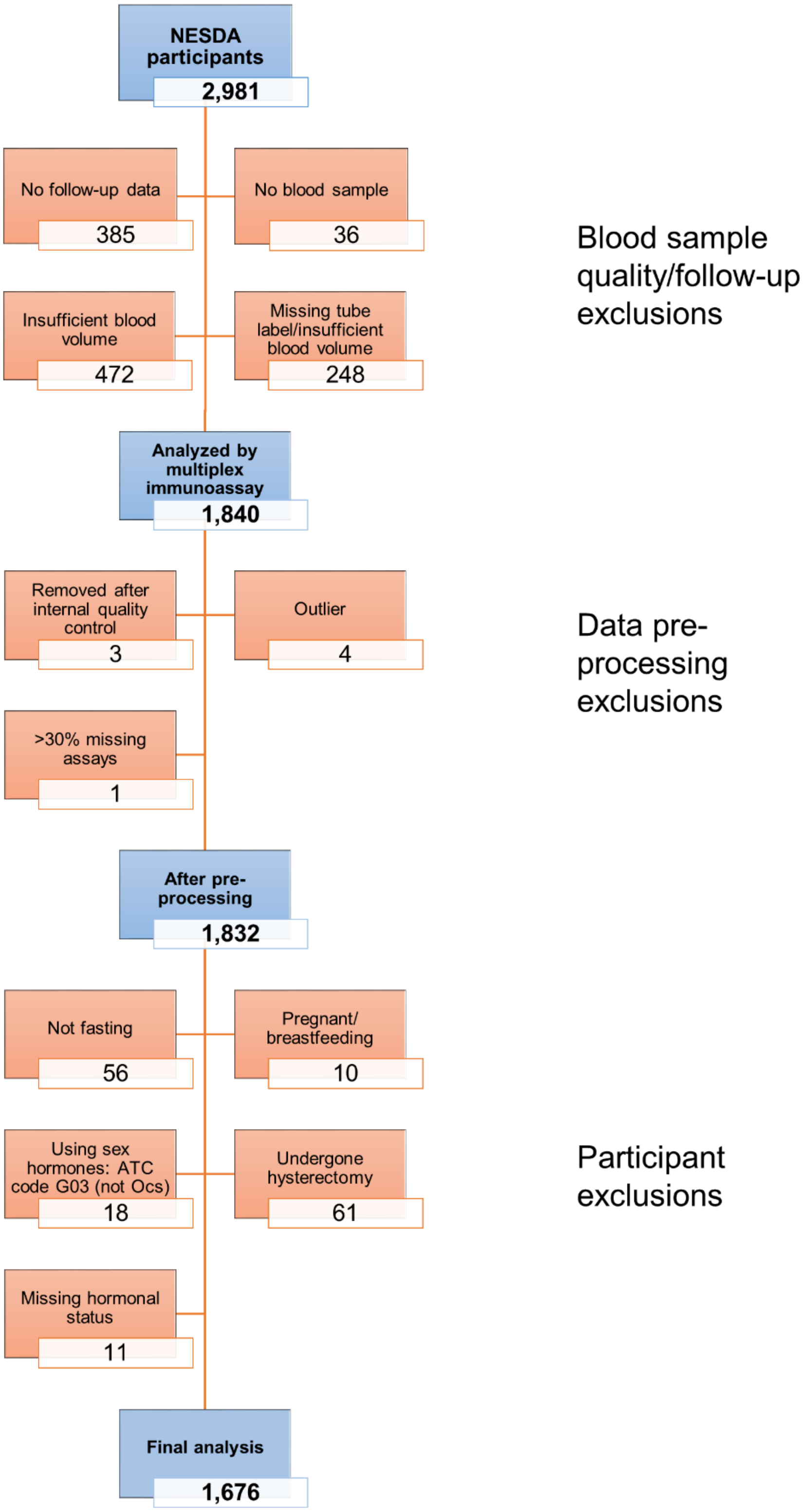

Supplementary Figure 2. Sample exclusions at each stage of analysis. NESDA samples were excluded due to quality of blood samples/data, lack of follow-up, and based on participant characteristics. Number of participants is indicated below each box. Orange box = excluded samples; blue box = samples remaining after exclusions at each stage. **Abbreviations:** NESDA (Netherlands Study of Depression and Anxiety); OC (oral contraceptive pill); HRT (hormone replacement therapy).

Supplementary Tables

Supplementary Table 1. List of molecules measured with multiplex immunoassay.

Analyte name	Short name	UniProtKB accession/ PubChem compound identifier	% missing	Analyzed
6Ckine		O00585	0	✓
α1-Antichymotrypsin	AACT	P01011	0	✓
α1-Antitrypsin	AAT	P01009	0.1	✓
α1-Microglobulin	A1Micro	P02760	0	✓
α2-Macroglobulin	A2Macro	P01023	0.2	✓
β2-Microglobulin	B2M	P61769	0.2	✓
Adiponectin		Q15848	0.2	✓
Agouti-Related Protein	AgRP	O00253	95.7	
Aldose Reductase		P15121	0.3	✓
Alpha-Fetoprotein	AFP	P02771	32.2	
Amphiregulin	AR	P15514		
Angiogenin		P03950	0.2	✓
Angiopoietin-2	ANG-2	O15123	0.1	✓
Angiotensin-Converting Enzyme	ACE	P12821	0	✓
Angiotensinogen		P01019	0.1	✓
Apolipoprotein A-I	Apo A-I	P02647	0	✓
Apolipoprotein A-II	Apo A-II	P02652	0	✓
Apolipoprotein A-IV	Apo A-IV	P06727	0	✓
Apolipoprotein B	Apo B	P04114	0	✓
Apolipoprotein C-I	Apo C-I	P02654	0	✓
Apolipoprotein C-III	Apo C-III	P02656	0	✓
Apolipoprotein D	Apo D	P05090	7.5	✓
Apolipoprotein E	Apo E	P02649	0.5	✓
Apolipoprotein H	Apo H	P02749	0.2	✓
Apolipoprotein(a)	Lp(a)	P08519	0.1	✓
AXL Receptor Tyrosine Kinase	AXL	P30530	0	✓
B Cell-Activating Factor	BAFF	Q9Y275	0	✓
B Lymphocyte Chemoattractant	BLC	O43927	94.4	
Betacellulin	BTC	P35070	99.0	
Brain Derived Neurotrophic Factor	BDNF	P23560	0.1	✓
Calbindin		P05937	99.3	
Cancer Antigen 125	CA 125	Q8WXI7	82.5	
Cancer Antigen 15-3	CA 15-3	P15941	0.1	✓
Cancer Antigen 19-9	CA 19-9	Q9BXJ9	84.4	
Cancer Antigen 72-4	CA 72-4		84.4	
Carcinoembryonic Antigen	CEA	P06731	0.2	✓
Cathepsin D		P07339	0.2	✓
CD40 Antigen	CD40	Q6P2H9	0.1	✓
CD40 Ligand	CD40L	P29965	0.4	✓
CD5 Antigen-Like	CD5L	O43866	0.1	✓
Cellular Fibronectin	cFib	P02751	28.7	✓
Chemokine CC-4	HCC-4	O15467	0	✓
Chromogranin A	CgA	P10645	2.2	✓
Ciliary Neurotrophic Factor	CNTF	P26441	99.9	
Clusterin	CLU	P10909	0.1	✓
Collagen IV		P02462 (a1), P08572 (a2), Q01955 (a3), P53420 (a4), P29400 (a5), Q14031 (a6)	0	✓
Complement C3	C3	P01024	0.1	✓
Complement Factor H – Related Protein 1	CFHR1	Q03591	0	✓
Cortisol		5754	0.2	✓
C-Peptide		P01308	0.3	✓
C-Reactive Protein	CRP	P02741	1.3	✓
Creatine Kinase-MB	CK-MB	P12277, P06732	6.1	✓
Cystatin-C		P01034	0	✓
E-Selectin		P16581	0.1	✓
Endoglin		P17813	0	✓
Endostatin		P39060	0.1	✓
EN-RAGE		P80511	0	✓
Eotaxin-1		P51671	11.6	✓
Eotaxin-2		O00175	0.3	✓
Eotaxin-3		Q9Y258	99.7	

Epidermal Growth Factor	EGF	P01133	0.3	✓
Epidermal Growth Factor Receptor	EGFR	P00533	0.1	✓
Epiregulin	EPR	O14944	99.0	
Epithelial Cell Adhesion Molecule	EpCam	P16422	98.6	
Epithelial-Derived Neutrophil-Activating Protein 78	ENA-78	P42830	0	✓
Ezrin		P15311	92.5	
Factor VII		P08709	0.1	✓
Fas Ligand	FasL	P48023	98.9	
FASLG Receptor	FAS	P25445	4.1	✓
Fatty Acid-Binding Protein, adipocyte	FABP, adipocyte	P15090	0.1	✓
Fatty Acid-Binding Protein, heart	FABP, heart	P05413	85.1	
Fatty Acid-Binding Protein, liver	FABP, liver	P07148	89.1	
Ferritin	FRTN	P02794, P02792	2.2	✓
Fetuin-A		P02765	0	✓
Fibrinogen		P02671, P02675, P02679	71.3	
Fibroblast Growth Factor 4	FGF-4	P08620	99.8	
Fibroblast Growth Factor basic	FGF-basic	P09038	99.5	
Fibulin-1C	Fib1C	P23142	0.4	✓
Follicle-Stimulating Hormone	FSH	P01225, P01215	2.0	✓
Galectin-3		P17931	0.1	✓
Gelsolin		P06396	0.2	✓
Glucagon		P01275	99.5	
Glucagon-Like Peptide 1, active	GLP-1 active		99.6	
Glucagon-Like Peptide 1, total	GLP-1 total	P01275	93.4	
Glucose-6-Phosphate Isomerase	G6PI	P06744	0	✓
Glutathione S-Transferase α	GSTA	P08263	26.1	✓
Glutathione S-Transferase Mu 1	GSTM1	P09488	95.3	
Granulocyte Colony-Stimulating Factor	G-CSF	P09919	32.0	
Granulocyte-Macrophage Colony-Stimulating Factor	GM-CSF	P04141	100	
Growth Hormone	GH	P01241	15.5	✓
Growth-Regulated α protein	GRO-A	P09341	0	✓
Haptoglobin		P00738	4.5	✓
HE4		Q14508	96.0	
Heat Shock Protein 60	HSP-60	P10809	99.4	
Heparin-Binding EGF-Like Growth Factor	HB-EGF	Q99075	31.3	
Hepatocyte Growth Factor	HGF	P14210	0.3	✓
Hepatocyte Growth Factor receptor	HGF receptor	P08581	0	✓
Hepsin		P05981	0	✓
Human Chorionic Gonadotropin β	hCGb	P01233	92.9	
Human Epidermal Growth Factor Receptor 2	HER-2	P04626	0	✓
Immunoglobulin A	IgA		0.1	✓
Immunoglobulin E	IgE		62.9	
Immunoglobulin M	IgM		0.2	✓
Insulin		P01308	20.6	✓
Insulin-like Growth Factor Binding Protein 1	IGFBP-1	P08833	1.4	✓
Insulin-like Growth Factor Binding Protein 2	IGFBP-2	P18065	0	✓
Insulin-like Growth Factor Binding Protein 3	IGFBP-3	P17936	0.1	✓
Insulin-like Growth Factor Binding Protein 4	IGFBP-4	P22692	0.1	✓
Insulin-like Growth Factor Binding Protein 5	IGFBP-5	P24593	0.1	✓
Insulin-like Growth Factor Binding Protein 6	IGFBP-6	P24592	0.1	✓
Interferon gamma	IFNγ	P01579	97.9	
Interferon-Inducible T-cell alpha chemoattractant	ITAC	O14625	30.4	
Intercellular Adhesion Molecule 1	ICAM-1	P05362	0.1	✓
Interferon γ Induced Protein 10	IP-10	P02778	0.6	✓
Interleukin-1 alpha	IL-1 alpha	P01583	30.3	
Interleukin-1 beta	IL-1 beta	P01584	94.2	
Interleukin-1 receptor antagonist	IL-1ra	P18510	1.6	✓
Interleukin-2	IL-2	P60568	100	
Interleukin-2 receptor α	IL-2RA	P01589	0	✓
Interleukin-3	IL-3	P08700	100	
Interleukin-4	IL-4	P05112	99.9	
Interleukin-5	IL-5	P05113	100	
Interleukin-6	IL-6	P05231	99.3	
Interleukin-6 receptor	IL-6r	P08887	0.1	✓
Interleukin-6 receptor subunit β	IL-6rB	P40189	0.2	✓
Interleukin-7	IL-7	P13232	98.5	
Interleukin-8	IL-8	P10145	13.1	✓
Interleukin-10	IL-10	P22301	92.3	
Interleukin-12 Subunit p70	IL-12p70	P29459	98.6	
Interleukin-12 Subunit p40	IL-12p40	P29460	3.5	✓
Interleukin-13	IL-13	P35225	99.3	
Interleukin-15	IL-15	P40933	76.9	
Interleukin-16	IL-16	Q14005	0.2	✓
Interleukin-17	IL-17	Q16552	32.1	
Interleukin-18	IL-18	Q14116	0.7	✓

Interleukin-23	IL-23	Q9NPF7	4.8	✓
Kallikrein 5		Q9Y337	0.8	✓
Kallikrein 7	KLK-7	P49862	99.6	
Kidney Injury Molecule-1	KIM-1	Q96D42	65.0	
Lactoylglutathione lyase	LGL	Q04760	0.1	✓
Latency-Associated Peptide of Transforming Growth Factor β 1	LAP TGF-b1	P01137	0	✓
Lectin-Like Oxidized LDL Receptor 1	LOX-1	P78380	28.7	✓
Leptin		P41159	0.2	✓
Luteinizing Hormone	LH	P01229, P01215	28.4	✓
Macrophage Colony-Stimulating Factor 1	M-CSF	P09603	3.8	✓
Macrophage Derived Chemokine	MDC	O00626	0	✓
Macrophage Inflammatory Protein-1 α	MIP-1A	P10147	90.6	
Macrophage Inflammatory Protein-3 α	MIP-3A	P78556	81.9	
Macrophage Inflammatory Protein-1 β	MIP-1B	P13236	0.1	✓
Macrophage Inflammatory Protein-3 β	MIP-3B	Q99731	0	✓
Macrophage Migration Inhibitory Factor	MIF	P14174	0.1	✓
Macrophage Stimulating Protein	MSP	P26927	0.2	✓
Malondialdehyde-Modified Low-Density Lipoprotein	MDA-LDL		97.5	
Maspin		P36952	99.9	
Matrix Metalloproteinase-1	MMP-1	P03956	0.9	✓
Matrix Metalloproteinase-3	MMP-3	P08254	0.1	✓
Matrix Metalloproteinase-7	MMP-7	P09237	0.2	✓
Matrix Metalloproteinase-9		P14780	88.0	
Matrix Metalloproteinase-9 (total)	MMP-9	P14780	0.2	✓
Matrix Metalloproteinase-10	MMP-10	P09238	0.2	✓
Mesothelin	MSLN	Q13421	0.1	✓
MHC Class I Chain-Related Protein A	MICA	Q29983	82.9	
Monocyte Chemotactic Protein 1	MCP-1	P13500	0.3	✓
Monocyte Chemotactic Protein 2	MCP-2	P80075	0.4	✓
Monocyte Chemotactic Protein 3	MCP-3	P80098	99.8	
Monocyte Chemotactic Protein 4	MCP-4	Q99616	2.0	✓
Monokine Induced by γ Interferon	MIG	Q07325	0.3	✓
Myeloid Progenitor Inhibitory Factor 1	MPIF-1	P55773	0.2	✓
Myeloperoxidase	MPO	P05164	1.7	✓
Myoglobin		P02144	0.2	✓
Nerve Growth Factor beta	NGF-beta	P01138	100	
Neuron Specific Enolase	NSE	P09104	0	✓
Neuronal Cell Adhesion Molecule	NrCAM	Q92823	2.0	✓
Neuropilin-1		O14786	0	✓
Neutrophil Gelatinase Associated Lipocalin	NGAL	P80188	0	✓
N-terminal prohormone of brain natriuretic peptide	NT proBNP	P16860	6.3	✓
Osteopontin		P10451	16.3	✓
Osteoprotegerin	OPG	O00300	0	✓
Pancreatic Polypeptide	PPP	P01298	0.1	✓
Pepsinogen I	PGI	O95576	0.2	✓
Peptide YY	PYY	P10082	70.7	
Phosphoserine Aminotransferase	PSAT	Q9Y617	0.1	✓
Placenta Growth Factor	PLGF	P49763	82.7	
Plasminogen Activator Inhibitor 1	PAI-1	P05121	0.2	✓
Platelet-Derived Growth Factor BB	PDGF-BB	P01127	0.1	✓
Progesterone		5994	19.9	✓
Proinsulin, intact		P01308	99.3	
Proinsulin, total		P01308	99.2	
Prolactin	PRL	P01236	0	✓
Prostasin		Q16651	0.1	✓
Prostate Specific Antigen, free	PSA-f	P07288	67.9	
Protein S100-A4	S100-A4	P26447	43.3	
Pulmonary and Activation-Regulated Chemokine	PARC	P55774	0	✓
Receptor for advanced glycosylation end products	RAGE	Q15109	0.1	✓
Receptor tyrosine-protein kinase erbB-3	ErbB3	P21860	0.1	✓
Resistin		Q9HD89	0.1	✓
S100 Calcium-Binding Protein-B	S100-B	P04271	97.9	
Serotransferrin	Transferrin	P02787	0	✓
Serum Amyloid P-Component	SAP	P02743	0.1	✓
Sex Hormone-Binding Globulin	SHBG	P04278	0.4	✓
Sortilin		Q99523	0.1	✓
Squamous Cell Carcinoma Antigen-1	SCCA-1	P29508	70.9	
Stem Cell Factor	SCF	P21583	0.5	✓
Stromal cell derived factor 1	SDF-1	P48061	0	✓
Superoxide Dismutase 1, soluble	SOD-1	P00441	0.8	✓
T-Cell-Specific Protein RANTES	RANTES	P13501	0.3	✓
T Lymphocyte-Secreted Protein I-309	I-309	P22362	40.3	
Tamm-Horsfall Urinary Glycoprotein	THP	P07911	0	✓
Tenascin-C	TN-C	P24821	0.1	✓
Testosterone (total)		6013	10.0	✓

Tetranectin		P05452	0.2	✓
Thrombomodulin	TM	P07204	0	✓
Thrombospondin-1		P07996	0	✓
Thyroglobulin	TG	P01266	25.5	✓
Thyroid Stimulating Hormone	TSH	P01215, P01222	0.4	✓
Thyroxine-Binding Globulin	TBG	P05543	0.2	✓
Tissue Inhibitor of Metalloproteinases 1	TIMP-1	P01033	0.2	✓
Tissue type Plasminogen activator	tPA	P00750	2.8	✓
TNF-Related Apoptosis-Inducing Ligand Receptor 3	TRAIL-R3	O14798	0	✓
Transforming Growth Factor alpha	TGF-alpha	P01135	96.2	
Transforming Growth Factor beta-3	TGF-beta-3	P10600	99.7	
Transthyretin	TTR	P02766	0	✓
Trefoil Factor 3	TFF3	Q07654	0	✓
Tumor Necrosis Factor alpha	TNF-alpha	P01375	99.6	
Tumor Necrosis Factor beta	TNF-beta	P01374	98.7	
Tumor necrosis Factor Receptor 2	TNFR2	P20333	0.2	✓
Tumor Necrosis Factor Receptor I	TNFRI	P19438	0	✓
Tyrosine kinase with Ig and EGF homology domains 2	TIE-2	Q02763	0.1	✓
Urokinase-type Plasminogen Activator	uPA	P00749	0.1	✓
Urokinase-type Plasminogen Activator Receptor	uPAR	Q03405	0.6	✓
Vascular Cell Adhesion Molecule 1	VCAM-1	P19320	0.2	✓
Vascular Endothelial Growth Factor	VEGF	P15692	0.1	✓
Vascular Endothelial Growth Factor B	VEGF-B	P49765	100	
Vascular Endothelial Growth Factor C	VEGF-C	P49767	0.1	✓
Vascular Endothelial Growth Factor D	VEGF-D	O43915	92.4	
Vascular Endothelial Growth Factor Receptor 1	VEGFR-1	P17948	99.6	
Vascular Endothelial Growth Factor Receptor 2	VEGFR-2	P35968	0	✓
Vascular Endothelial Growth Factor Receptor 3	VEGFR-3	P35916	1.0	✓
Vitamin D-Binding Protein	VDBP	P02774	0.1	✓
Vitamin K-Dependent Protein S	VKDPS	P07225	0	✓
Vitronectin		P04004	0.1	✓
von Willebrand Factor	vWF	P04275	1.1	✓
YKL-40		P36222	0.2	✓

Supplementary Table 2. Description of variables. It should be noted that medication use was evaluated by self-reporting and inspection of drug containers used in the past month. **Abbreviations:** ATC (Anatomical Therapeutic Chemical)

**Variable**	**Measurement units (continuous) or categories**	**Additional information**
Age	Years	
Body mass index (BMI)	kg/m^2	
Waist circumference	Centimetres	
Ancestry	North European or other	
Collection area	Amsterdam, Leiden, or Groningen	
Recruitment method	Community, general practice, secondary mental health care	
Education	Years	
Partner status	Yes or no	
Smoking status	Never smoked, former smoker, regular smoker, or not a regular smoker	
Alcohol consumption	Drinks/week	
Recreational drug use in the past month	Yes or no	Recreational drugs considered were cannabis, speed, cocaine, heroin, and LSD.
Presence of treated chronic somatic disease	Yes or no	*Chronic diseases considered were cardiovascular disease, diabetes, lung disease, osteoarthritis, rheumatic disease, cancer, ulcer, intestinal problem, liver disease, epilepsy, thyroid gland disease and others. Presence of cardiovascular disease was assessed by self-report and confirmed by use of appropriate medication, while presence of diabetes was assessed by use of anti-diabetic medication (ATC code A10) or by fasting plasma glucose level ≥7.0 mmol l^{-1}. Other chronic diseases were treated and assessed by self-report.*
Use of anti-inflammatory drugs	Yes or no	ATC codes H02, R03BA, R03AK, D07, M01A, M01B, A07EB or A07EC
Use of lipid modifying agents	Yes or no	ATC code C10
Use of antihypertensive medication	Yes or no	ATC codes C02, C03, C07, C08, or C09
Physical activity	Metabolic equivalent (MET)/minute	*Assessed with the International Physical Activity Questionnaire*
Blood pressure	mm Hg	*Systolic and diastolic blood pressure measured*
Depressive symptoms	30-item self-rated Inventory of Depressive Symptomatology (IDS) score	
Sex	Male, female	
Hormonal status	Male or female in the follicular phase of the menstrual cycle, luteal phase, using oral contraceptives, after menopause, and others (see Additional Information).	*Assessed by self-report. Use of oral contraceptives and sex hormones (ATC code G03) was assessed by self-report. Classification of menstrual cycle phases in women [follicular (0-13 days) and luteal (14-32 days or more)], postmenopausal status (yes/no), hysterectomized (yes/no), and pregnant or breastfeeding (yes/no) were also self-reported.*

Supplementary Table 3. Demographic, lifestyle, and health characteristics for NESDA (A) discovery samples and (B) validation samples. The set of subjects in **(A)** was used to test for variations in serum analyte levels with sex and female hormonal status and to construct random forest classifiers to group subjects as males, oral contraceptive (OC) users, postmenopausal females, and females with a menstrual cycle. The set of subjects in **(B)** was used to validate our findings and as a test set for the random forest classifiers built using the discovery cohort. Values are shown as the mean ± the standard deviation. Differences between groups were assessed using ANOVA (continuous data) or Fisher's exact test (categorical data). Variables in bold are significantly different (significance defined as $p < 0.05$) between groups. **Abbreviations**: BMI (body mass index); IDS (Inventory of Depressive Symptomatology); OC (oral contraceptive pill).

**(A)**	**Female**				**Male**
	**OC user**	**Follicular phase**	**Luteal phase**	**Postmenopausal**	
N	79	34	37	57	140
**Age (years)**	28±10	36.4±12	35.8±10	55.9±6	39.7±15
**BMI (kg/m^2)**	23.4±4	25.1±6	25.1±5	25.8±5	25.3±4
**Waist circumference (cm)**	79.5±12	82.5±13	85.4±14	88.3±12	93.1±13
North European ancestry % (Yes/No)	96/4	94/6	100/0	98/2	99/1
**Area % (Amsterdam/Groningen/Leiden)**	10/68/22	15/38/47	22/41/38	26/23/51	11/51/38
**Frame % (General population/Primary care)**	48/52	29/71	27/73	0/100	33/67
Education (years)	12.6±3	13.1±4	13.7±3	11.9±4	12.7±3
Partner % (Yes/No)	73/27	79/21	76/24	82/18	74/26
Smoking % (Current smoker/Former smoker/Never smoker)	19/30/51	32/35/32	30/19/51	23/44/33	31/37/32
**Alcohol (drinks per week)**	4.4±5	2.8±5	5.5±7	6.6±8	10.8±11
Recreational drug use in last month % (Yes/No)	5/95	9/91	3/97	0/100	9/91
**Chronic disease % (Yes/No)**	19/81	38/62	22/78	51/49	31/69
Anti-inflammatory medication % (Yes/No)	3/97	3/97	5/95	4/96	1/99
Lipid modifying agents % (Yes/No)	1/99	0/100	0/100	7/93	8/92
**Antihypertensive medication % (Yes/No)**	9/91	3/97	3/97	23/77	15/85
Physical activity (MET)	3822.4±2571	4616.2±4093	3079.5±2595	3626.2±2687	4154.5±3551
**Systolic blood pressure (mm Hg)**	127.6±15	122.5±12	124.7±14	139.5±28	144.8±18
**Diastolic blood pressure (mm Hg)**	77.3±10	74.4±8	76.8±11	80.7±15	81.7±12
**IDS score**	8.5±7	8.8±9	7.3±6	9.8±8	5.5±5

**(B)**	**Female**				**Male**
	**OC user**	**Follicular phase**	**Luteal phase**	**Postmenopausal**	
N	263	149	211	261	445
**Age (years)**	30.9±10	36.2±9	36.5±9	55±6	43.4±13
**BMI (kg/m^2)**	24.4±5	25.3±5	25.3±6	26.9±5	26.3±5
**Waist circumference (cm)**	81.8±12	84.7±13	84.4±14	90.7±13	96.2±14
North European ancestry % (Yes/No)	95/5	97/3	94/6	94/6	97/3
**Area % (Amsterdam/Groningen/Leiden)**	17/44/38	22/38/40	25/42/33	16/38/46	16/42/42
**Frame % (General population/Primary care)**	25/39/36	18/48/34	15/44/41	12/72/16	20/42/38
**Education (years)**	11.9±3	12.3±3	12.3±3	11.5±3	11.8±3
Partner % (Yes/No)	69/31	66/34	67/33	70/30	72/28
**Smoking % (Current smoker/Former smoker/Never smoker)**	37/27/37	36/28/36	46/29/25	29/46/25	43/34/23
**Alcohol (drinks per week)**	4±6	4.8±8	5.6±9	6.3±8	11±14
**Recreational drug use in last month % (Yes/No)**	10/90	5/95	7/93	2/98	9/91
**Chronic disease % (Yes/No)**	35/65	33/67	33/67	60/40	47/53
Anti-inflammatory medication % (Yes/No)	6/94	7/93	3/97	8/92	6/94
**Lipid modifying agents % (Yes/No)**	1/99	2/98	1/99	12/88	11/89
**Antihypertensive medication % (Yes/No)**	3/97	6/94	7/93	27/73	18/82
Physical activity (MET)	3763.3±3264	3703.6±2772	3340.5±2920	3749.2±2908	3728.3±3241
**Systolic blood pressure (mm Hg)**	126.8±13	126.7±17	124.9±19	143.1±22	144.1±20
**Diastolic blood pressure (mm Hg)**	77.8±9	79.6±11	77.7±13	85.6±11	84.5±12
IDS score	23.6±12	24.4±13	24.7±13	24±13	24.5±13

Supplementary Table 4. Serum analytes elevated in (A) females and (B) males. Analytes are ordered from top to bottom by decreasing significance in the discovery cohort (reported in the columns of *q*-values). Robust regression was used where * follows the analyte name. The $\log_2$ ratios of serum molecular concentrations in males compared to females were coefficients from the linear regression. Serum analytes that varied significantly with female hormonal status are indicated (see **Supplementary Table 5**). These are coloured in blue where analytes were significantly different between oral contraceptive pill users and females with a menstrual cycle; orange where they were significantly different between postmenopausal females and females with a menstrual cycle; and yellow where there were multiple significant contrasts. Agreement with the validation cohort and our previous study is shown by a ✓. SCZ, MDD, and cancer markers are indicated by ↑ (elevated in patients); ↓ (reduced in patients); or ✕ (conflicting evidence for elevated and reduced levels in patients). Biological processes were found from gene ontology (GO) terms. **Abbreviations:** NM (not measured in the previous study); SCZ (schizophrenia); MDD (major depressive disorder); MP (metabolic process); DP (developmental process); CC & ST (cell communication and signal transduction); IR (defense/immune/inflammatory response); CP (cell proliferation); NSD (nervous system development); VD (vasculature development). Analyte abbreviations can be found in **Supplementary Table 1**. **GO terms (left to right)**: GO:0008152; GO:0032502; GO:0007154 and GO:0007165; GO:0006810; GO:0040007 and GO:0008283; GO:0006935; GO:0006950; GO:0008219; GO:0006952 and GO:0006955 and GO:0006954; GO:0007399; GO:0001944.

(A)									Biological processes										
Analytes	$\log_2$ ratio (male/female)	Q-value	Varies with hormonal status	Agrees with *Ramsey et al, 2012*	Agrees with test cohort	SCZ marker	MDD marker	Cancer marker	MP	DP	CC & ST	Transport	Growth/CP	Chemotaxis	Response to stress	Cell death	IR	NSD	VD
Leptin	-2.06	4.E-64		✓	✓	↑	↑	↓	✓	✓	✓	✓	✓		✓	✓		✓	
FABP, adipocyte*	-0.68	1.E-31	✓	NM	✓				✓	✓					✓		✓		
GH	-2.38	1.E-29	✓	✓	✓	↓	↓	↑	✓	✓	✓	✓	✓						
SHBG	-1.09	4.E-29	✓	✓	✓					✓			✓						
Apo A-I*	-0.25	3.E-17		✓	✓	↓	↓	↓	✓	✓	✓	✓	✓	✓	✓		✓	✓	
Adiponectin	-0.60	8.E-15		✓	✓	↓	↓		✓	✓	✓	✓	✓		✓	✓	✓		
TBG	-0.29	6.E-11	✓	✓	✓	↑			✓	✓		✓							
IGFBP-1	-1.04	1.E-10	✓	NM	✓			↑	✓	✓	✓		✓		✓				
FSH*	-0.99	1.E-10	✓		✓	↑		↓	✓	✓	✓		✓						
CRP	-1.16	4.E-10	✓	✓	✓	↑	↑	↑	✓	✓		✓			✓		✓	✓	
uPAR*	-0.37	1.E-09		NM	✓			↑	✓		✓			✓	✓				
ErbB3*	-0.52	3.E-09	✓	NM	✓				✓	✓	✓	✓	✓		✓	✓	✓	✓	
NT proBNP	-0.91	5.E-09	✓	✓	✓				✓	✓	✓	✓	✓						✓
OPG*	-0.23	1.E-08	✓	NM	✓			↑		✓	✓					✓			
AAT*	-0.20	1.E-07	✓	✓	✓	↑		↑	✓			✓			✓		✓		
A2Macro	-0.21	2.E-07		✓	✓		↓	✕	✓	✓	✓	✓			✓		✓		
PRL*	-0.36	7.E-07			✓			↑		✓	✓	✓	✓						
ENA- 78*	-0.41	1.E-06		✓	✓	↑	↓	↓			✓		✓	✓	✓		✓		
BAFF*	-0.15	3.E-06		NM	✓			↑			✓	✓	✓				✓		
Factor VII*	-0.17	2.E-05	✓	✓	✓	✕		↑	✓	✓	✓			✓	✓				
CLU*	-0.09	6.E-05	✓		✓			↓	✓	✓	✓	✓	✓		✓	✓	✓	✓	
TFF3*	-0.17	1.E-04	✓	✓	✓										✓		✓		
Transferrin	-0.11	3.E-04	✓	✓	✓	↓	↓	↓				✓			✓				
IgM	-0.27	7.E-04	✓	✓	✓		↓												
VEGFR-3*	-0.27	8.E-04	✓	NM				↓	✓	✓	✓		✓		✓	✓			✓
GRO-A*	-0.24	0.001		✓	✓				✓	✓	✓		✓	✓	✓		✓	✓	
Fetuin A	-0.12	0.002	✓	✓		↓		↓	✓	✓	✓	✓			✓		✓		
Collagen IV	-0.23	0.002	✓	NM	✓				✓	✓	✓	✓	✓	✓		✓		✓	✓
MMP-7	-0.12	0.003		✓	✓			↑	✓			✓	✓		✓		✓		
Galectin-3*	-0.11	0.003		NM	✓				✓	✓	✓	✓	✓	✓	✓	✓	✓		
MDC	-0.38	0.004			✓	↑		↓			✓			✓	✓		✓		
Haptoglobin	-0.14	0.004		✓	✓	↑		↑	✓			✓			✓	✓	✓		
THP*	-0.17	0.005		✓	✓					✓			✓		✓		✓		
TSH*	-0.23	0.008					↓		✓		✓		✓						
TG*	-0.27	0.013		NM	✓			↑	✓	✓	✓	✓						✓	
uPA	-0.13	0.016	✓	NM	✓				✓		✓		✓	✓	✓				
Apo A-II*	-0.08	0.016	✓	NM	✓	↓		↑	✓	✓	✓	✓			✓		✓		
LOX-1*	-0.20	0.018	✓	✓					✓						✓	✓	✓		
M-CSF*	-0.15	0.018	✓					✕	✓	✓	✓		✓		✓		✓		
Vitronectin	-0.08	0.029	✓	✓	✓			↑	✓	✓	✓	✓			✓		✓		
Resistin	-0.15	0.032			✓	↓			✓	✓	✓	✓	✓						
IL-12p40*	-0.10	0.049		✓		↓			✓	✓	✓	✓	✓		✓	✓	✓		
C-Peptide*	-0.11	0.050	✓					↑	✓	✓	✓	✓	✓		✓	✓	✓		

(B)

Analytes	$\log_2$ ratio (male/female)	Q-value	Varies with female hormonal status	Agrees with *Ramsey et al, 2012*	Agrees with test cohort	SCZ marker	MDD marker	Cancer marker	Biological processes: MP	DP	CC & ST	Transport	Growth/CP	Chemotaxis	Response to stress	Cell death	IR	NSD	VD
Testosterone*	1.98	3.E-174	✓	✓	✓														
MMP- 3	0.99	2.E-46		✓	✓			↑	✓		✓				✓	✓			
FRTN	1.65	4.E-27	✓	✓	✓	↑	↑	↑				✓	✓				✓		
HER- 2*	0.38	1.E-14	✓	NM	✓			↑	✓	✓	✓		✓	✓	✓		✓	✓	✓
Eotaxin-1	0.58	8.E-13		✓	✓				✓	✓	✓		✓	✓	✓		✓		✓
AXL*	0.33	5.E-10	✓	✓	✓				✓	✓	✓	✓			✓	✓	✓	✓	
VKDPS*	0.19	3.E-09		✓	✓				✓			✓			✓		✓		
tPA	0.33	4.E-09	✓	NM	✓				✓		✓	✓			✓				
NrCAM	0.40	4.E-09		NM	✓	↓				✓	✓		✓	✓				✓	✓
Myoglobin*	0.34	1.E-08		✓	✓					✓					✓				
TM*	0.20	1.E-08	✓	✓	✓										✓				
TTR	0.17	1.E-07	✓	✓	✓	↓		↓	✓		✓	✓							
PSAT*	0.43	2.E-07		NM	✓				✓										
E-Selectin	0.35	4.E-07	✓	✓	✓			✗	✓		✓	✓			✓		✓		
IL-6rB*	0.11	9.E-07	✓	NM	✓			↑	✓	✓	✓		✓		✓	✓	✓	✓	
Osteopontin	0.41	1.E-06	✓	✓	✓			↑		✓			✓	✓	✓		✓	✓	
CK-MB	0.45	2.E-06		✓	✓				✓	✓								✓	
IGFBP-6*	0.17	4.E-06		NM	✓				✓		✓		✓						
SAP*	0.21	5.E-06	✓	✓	✓	↑	↑		✓	✓					✓		✓		
Apo D	0.18	6.E-06	✓	✓	✓	✗			✓	✓	✓	✓	✓		✓		✓	✓	✓
HCC-4*	0.29	1.E-05	✓	✓	✓			↑			✓			✓	✓		✓		
Prostasin*	0.22	2.E-05		NM	✓				✓			✓							
Angiogenin	0.15	7.E-05	✓	NM	✓			↑	✓	✓	✓	✓	✓		✓				✓
ALDR*	0.14	8.E-05		NM	✓				✓						✓				
Cathepsin D	0.16	1.E-04		NM	✓			↑	✓										
HGF	0.26	1.E-04			✓				✓	✓	✓	✓	✓	✓	✓	✓		✓	✓
FAS*	0.22	2.E-04		✓	✓			↑	✓	✓	✓				✓	✓	✓		
Eotaxin-2	0.39	2.E-04		NM	✓				✓	✓	✓		✓	✓	✓		✓		✓
Apo H*	0.14	2.E-04	✓	✓	✓	↑			✓	✓		✓	✓		✓	✓			✓
PGI*	0.17	4.E-04		NM	✓				✓										
MIP-1B	0.26	5.E-04	✓	✓	✓	↑	↑	↑	✓		✓	✓		✓	✓		✓		
A1Micro	0.10	5.E-04		✓	✓				✓		✓	✓			✓		✓		
IL-18	0.26	5.E-04			✓	↑			✓	✓	✓	✓	✓		✓		✓		✓
ACE	0.16	6.E-04	✓		✓		↓	↓	✓	✓		✓	✓						
CEA*	0.35	6.E-04		✓	✓	↑		↑		✓						✓			
MCP-1*	0.21	7.E-04		✓	✓	↑		↑	✓	✓	✓	✓	✓	✓	✓	✓	✓	✓	✓
LGL*	0.27	8.E-04		NM	✓				✓	✓						✓			
Gelsolin	0.12	0.001		NM	✓					✓	✓	✓	✓		✓	✓		✓	
CA 15-3	0.26	0.002		NM	✓			↑	✓		✓				✓	✓			
GSTA	0.44	0.002			✓	↑			✓	✓									
NSE*	0.21	0.003		NM	✓			↑	✓										
IL-6r	0.14	0.005			✓	↑		↑	✓	✓	✓		✓	✓	✓	✓	✓		
MCP-4	0.18	0.008	✓	✓	✓					✓	✓			✓	✓		✓		
Cystatin-C	0.06	0.018		✓	✓			↓	✓	✓					✓		✓		
CgA*	0.17	0.021	✓		✓	↑		↑											
SCF*	0.09	0.022			✓	✗		✗	✓	✓	✓		✓		✓	✓	✓		
TSP1	0.12	0.023		✓	✓	↓		↑	✓	✓	✓	✓	✓	✓	✓	✓	✓		✓
6Ckine	0.07	0.024		NM	✓			↑	✓	✓	✓	✓		✓	✓	✓	✓		
MCP-2*	0.13	0.032			✓				✓		✓	✓		✓	✓		✓		
IGFBP-2	0.15	0.035	✓	✓	✓	↑		↑	✓	✓	✓		✓						
Tetranectin*	0.08	0.038		NM	✓				✓	✓									
SOD-1*	0.15	0.039		✓	✓		↑		✓	✓	✓	✓	✓		✓	✓		✓	
MIF*	0.18	0.043			✓	↑	↑	↑	✓	✓	✓	✓	✓	✓	✓	✓	✓		

Supplementary Table 5. Serum analytes varying significantly with female hormonal status. Analytes are ordered from top to bottom by decreasing significance in the discovery cohort (reported in the column of ANOVA *q*-values). Robust regression was used where * follows the analyte name. The $\log_2$ ratios of serum molecular concentrations were coefficients from the linear regression. Agreement with the validation cohort is shown by a ✓. SCZ, MDD, and cancer markers are indicated by ↑ (elevated in patients); ↓ (reduced in patients); or ✕ (conflicting evidence for elevated and reduced levels in patients). Biological processes were found from gene ontology (GO) terms. Significant differences in analyte concentrations between groups are highlighted. **Abbreviations:** Q (*q*-value); R ($\log_2$ ratio); SCZ (schizophrenia); MDD (major depressive disorder); MP (metabolic process); DP (developmental process); CC & ST (cell communication and signal transduction); IR (defense/immune/inflammatory response); CP (cell proliferation); NSD (nervous system development); VD (vasculature development). Analyte abbreviations can be found in **Supplementary Table 1**. **GO terms (left to right):** GO:0008152; GO:0032502; GO:0007154 and GO:0007165; GO:0006810; GO:0040007 and GO:0008283; GO:0006935; GO:0006950; GO:0008219; GO:0006952 and GO:0006955 and GO:0006954; GO:0007399; GO:0001944.

	ANOVA	Follicular phase / luteal phase		Oral contraception / menstrual cycle		Postmenopausal / menstrual cycle		Males / females with menstrual cycle		Agrees with test cohort	SCZ marker	MDD marker	Cancer marker	Biological processes										
														MP	DP	CC & ST	Transport	Growth / CP	Chemotaxis	Response to stress	Cell death	IR	NSD	VD
Analytes	Q	R	Q	R	Q	R	Q	R	Q															
LH*	3.E-135	0.27	0.17	-0.46	3.E-06	2.03	6.E-39	-0.24	0.024	✓	↑		↓	✓	✓	✓								
FSH*	2.E-78	0.69	7.E-04	-0.93	4.E-09	2.68	2.E-39	-0.50	4.E-05	✓	↑		↓	✓	✓	✓		✓						
TFF3*	2.E-65	0.10	0.32	3.43	2.E-48	0.01	0.97	-0.15	0.008	✓										✓		✓		
TBG	8.E-24	0.01	0.98	0.48	1.E-18	-0.18	0.008	-0.16	0.001	✓	↑			✓	✓		✓							
AAT*	8.E-20	0.14	0.055	0.43	1.E-16	-0.03	0.78	-0.05	0.31	✓	↑		↑	✓			✓			✓		✓		
SHBG	1.E-16	0.21	0.32	0.86	8.E-12	-0.21	0.16	-0.83	1.E-13	✓					✓			✓						
ErbB3*	2.E-15	-0.32	0.12	0.91	1.E-11	0.11	0.54	-0.18	0.15	✓				✓	✓	✓	✓	✓		✓	✓	✓	✓	
Osteopontin	2.E-15	0.14	0.46	-0.61	1.E-07	0.64	7.E-06	0.38	2.E-04	✓			↑		✓			✓	✓	✓		✓	✓	
SAP*	1.E-14	-0.03	0.85	0.49	2.E-14	0.18	0.025	0.44	1.E-16	✓	↑	↑		✓	✓					✓		✓		
Testosterone*	4.E-13	-0.11	0.24	-0.32	2.E-08	0.18	0.012	1.91	2.E-137	✓														
Progesterone	6.E-12	-0.72	5.E-08	-0.44	2.E-06	-0.27	0.020	-0.22	0.010	✓	↑													
Transferrin	3.E-11	-0.08	0.25	0.27	5.E-11	-0.02	0.81	-0.01	0.85	✓	↓	↓	↓				✓			✓				
IGFBP-2	1.E-10	0.33	0.025	-0.65	8.E-11	-0.11	0.43	-0.12	0.24	✓	↑		↑	✓	✓	✓		✓						
HER-2*	9.E-10	-0.02	0.95	-0.34	2.E-06	0.28	0.002	0.34	4.E-08	✓			↑	✓	✓	✓		✓	✓	✓		✓	✓	✓
CRP	4.E-09	0.22	0.66	1.45	4.E-08	-0.06	0.91	-0.64	0.008	✓	↑	↑	↑	✓	✓		✓			✓		✓	✓	
Vitronectin	4.E-09	0.06	0.51	0.29	3.E-08	-0.07	0.33	0.00	0.98	✓			↑	✓	✓	✓	✓			✓		✓		
Factor VII*	5.E-07	0.02	0.89	0.33	4.E-08	0.16	0.017	0.00	0.99	✓	✕		↑	✓	✓	✓			✓	✓				
TTR	7.E-07	-0.13	0.056	0.23	1.E-07	0.13	0.006	0.30	2.E-13	✓	↓		↓	✓		✓	✓							
MCP-4	4.E-06	0.03	0.89	-0.39	7.E-05	0.22	0.072	0.10	0.30	✓					✓	✓			✓	✓		✓		
OPG*	5.E-06	0.14	0.11	0.34	1.E-06	0.09	0.19	-0.09	0.11	✓			↑		✓	✓					✓			
CLU*	6.E-06	0.02	0.78	0.18	1.E-06	0.11	0.014	0.00	0.99	✓			↓	✓	✓	✓	✓	✓		✓	✓	✓	✓	
C3	7.E-06	-0.01	0.98	0.24	3.E-07	0.09	0.081	0.10	0.017	✓	↑	↑		✓	✓	✓	✓			✓		✓		✓
Fetuin-A	8.E-06	-0.06	0.51	0.27	2.E-06	0.00	0.99	-0.02	0.81	✓	↓		↓	✓	✓	✓	✓			✓		✓		
M-CSF*	1.E-05	-0.13	0.32	0.43	3.E-06	0.04	0.78	0.02	0.85	✓			✕	✓	✓	✓		✓		✓		✓		
uPA	9.E-05	0.11	0.41	-0.34	2.E-05	-0.07	0.47	-0.28	7.E-05	✓				✓		✓		✓	✓	✓				
VEGFR-2*	2.E-04	-0.04	0.67	0.22	1.E-05	0.07	0.30	0.11	0.009	✓			↓	✓	✓	✓		✓	✓		✓			✓
CgA*	2.E-04	-0.09	0.64	-0.42	2.E-04	0.16	0.32	0.07	**0.51**	✓	↑		↑											

AXL*	2.E-04	0.15	0.30	-0.02	0.87	0.29	0.001	0.41	8.E-08	✓				✓	✓	✓	✓			✓	✓	✓	✓	
IGFBP-3*	2.E-04	0.08	0.034	-0.07	0.026	0.06	0.11	-0.03	0.31	✓			✕	✓	✓	✓		✓			✓			
VEGFR-3*	2.E-04	-0.12	0.52	0.47	5.E-05	0.01	0.98	-0.10	0.37	✓			↓	✓	✓	✓		✓		✓	✓			✓
Apo A-II*	3.E-04	-0.02	0.91	0.21	4.E-05	0.14	0.008	0.04	0.41	✓	↓		↑	✓	✓	✓	✓			✓		✓		
MSP*	3.E-04	0.00	1.00	0.39	1.E-04	0.28	0.004	0.18	0.017	✓					✓									
IgM	5.E-04	0.27	0.15	0.16	0.24	-0.43	0.003	-0.34	0.002	✓		↓												
Lp(a)	5.E-04	0.80	0.071	-0.87	0.004	0.23	0.54	-0.48	0.082			↑		✓			✓							
IGFBP-1	5.E-04	0.35	0.36	0.95	8.E-05	0.29	0.33	-0.59	0.006	✓			↑	✓	✓	✓		✓		✓				
VDBP	5.E-04	-0.23	0.15	0.40	2.E-04	0.04	0.85	0.07	**0.51**	✓				✓			✓							
MMP-9*	8.E-04	0.05	0.83	0.25	0.008	-0.12	0.28	0.03	0.85	✓	↑	↑	↑	✓	✓	✓	✓		✓		✓		✓	
VCAM-1	9.E-04	0.08	0.37	-0.17	0.002	0.02	0.80	0.00	0.99	✓	↑	↑	↑	✓	✓	✓		✓	✓	✓		✓		
tPA	0.001	0.00	0.99	-0.28	8.E-04	0.08	0.52	0.26	7.E-04	✓				✓		✓	✓			✓				
Fib 1C*	0.002	0.08	0.41	-0.15	0.007	0.04	0.55	0.00	0.99	✓					✓									
Neuropilin-1	0.002	0.18	0.021	-0.14	0.012	0.01	0.91	0.02	0.83	✓				✓	✓	✓	✓	✓	✓	✓	✓		✓	✓
Apo D	0.003	-0.11	0.24	-0.20	0.002	-0.16	0.026	0.06	0.32	✓	✕			✓	✓	✓	✓	✓		✓		✓	✓	✓
TM*	0.003	0.08	0.32	-0.09	0.091	0.15	0.025	0.21	4.E-06	✓										✓				
Insulin	0.005	0.01	0.99	0.62	4.E-04	0.23	0.27	0.29	0.082	✓	↑	↑	↑	✓	✓	✓	✓	✓		✓	✓	✓		
Angiogenin	0.005	0.01	0.98	0.20	6.E-04	0.14	0.063	0.27	1.E-07	✓			↑	✓	✓	✓	✓	✓		✓				✓
IGFBP-4*	0.005	0.00	0.99	0.17	5.E-04	0.10	0.11	0.03	0.61	✓				✓	✓	✓		✓		✓		✓		
HCC-4*	0.008	0.06	0.81	0.34	0.003	0.32	0.008	0.51	3.E-08	✓			↑			✓			✓	✓		✓		
ANG-2*	0.009	0.00	1.00	-0.39	0.002	-0.08	0.59	-0.17	0.11	✓	↑		↑		✓	✓			✓	✓				✓
NT proBNP	0.010	0.30	0.44	0.00	0.99	-0.87	0.002	-1.16	2.E-08	✓				✓	✓	✓	✓	✓						✓
Apo C-III	0.010	-0.15	0.25	0.24	0.003	0.19	0.055	0.07	0.40	✓	↑	↑	↓	✓		✓	✓			✓		✓		
IL-1ra	0.011	-0.06	0.66	0.23	0.002	-0.01	0.96	0.06	**0.41**	✓	↑	↑	↓	✓	✓	✓	✓			✓	✓	✓		
FRTN	0.013	-0.02	0.99	0.06	0.89	0.86	0.002	1.92	1.E-21	✓	↑	↑	↑				✓	✓				✓		
Angiotensinogen	0.013	0.22	0.84	1.44	0.002	0.24	0.74	0.25	0.64	✓				✓	✓	✓	✓	✓		✓	✓	✓	✓	✓
E-Selectin	0.015	-0.17	0.32	-0.28	0.009	0.00	0.99	0.25	0.009	✓			✕	✓		✓	✓			✓		✓		
Apo H*	0.016	0.00	0.99	0.07	0.30	0.23	0.002	0.24	1.E-05	✓	↑			✓	✓		✓	✓		✓	✓			✓
MIG*	0.016	0.22	0.28	0.33	0.012	0.27	0.11	0.15	0.22	✓				✓	✓	✓	✓	✓	✓	✓		✓		
HGF receptor*	0.020	0.01	0.96	0.17	0.006	0.14	0.025	0.09	0.056	✓				✓	✓	✓	✓	✓	✓	✓	✓		✓	
IL-6rB*	0.021	0.00	0.99	-0.02	0.62	0.12	0.010	0.14	2.E-05	✓			↑	✓	✓	✓		✓		✓	✓	✓	✓	
C-Peptide*	0.023	0.01	0.98	0.23	0.003	0.14	0.15	0.02	0.89	✓			↑	✓	✓	✓	✓	✓		✓	✓	✓		
LOX-1*	0.024	-0.01	0.99	0.17	0.25	-0.25	0.064	-0.21	0.055	✓				✓						✓	✓	✓		
ACE	0.031	-0.10	0.41	-0.10	0.20	0.13	0.14	0.16	0.017	✓		↓	↓	✓	✓		✓	✓						
GH	0.031	-0.13	0.86	0.68	0.032	-0.24	0.55	-2.19	3.E-16	✓	↓	↓	↑	✓	✓	✓	✓	✓						
SDF-1*	0.032	-0.04	0.52	-0.10	0.016	-0.10	0.025	-0.04	0.25	✓					✓	✓	✓	✓	✓	✓	✓	✓	✓	✓
MIP-1B	0.034	0.27	0.13	-0.26	0.026	0.04	0.85	0.18	0.10	✓	↑	↑	↑	✓		✓	✓		✓	✓		✓		
FABP, adipocyte*	0.037	0.16	0.19	0.12	0.22	0.24	0.020	-0.57	4.E-16					✓	✓					✓		✓		
Collagen IV	0.039	0.32	0.064	-0.15	0.23	-0.27	0.034	-0.37	3.E-04	✓				✓	✓	✓	✓	✓	✓		✓		✓	✓

Supplementary Table 6. Accuracy of random forest classifiers predicting sex and female hormonal status using all data for the (A) discovery cohort and (B) validation cohort and (C) using only the ten most important variables for the validation cohort. Accuracies of predictions using the first classifier (constructed using all analyte and covariate data; 189 total predictors) are shown for the **(A)** 347 discovery samples (out-of-bag (OOB) accuracy is shown) and **(B)** 1,329 NESDA validation samples. Accuracies of predictions using the second classifier (constructed with discovery cohort data using only the ten most important variables for classification) is shown in **(C)** for the 1,329 NESDA validation samples.

Actual class (discovery cohort; % classified)	(A)	*Classifier constructed using all data*				Discovery accuracy (%)
		Predicted class (discovery cohort)				
		OC user	Menstrual cycle	Postmeno-pausal	Male	
	OC user	**73 (92.4%)**	5 (6.3%)	1 (1.3%)	0 (0%)	92.4
	Menstrual cycle	3 (4.2%)	**62 (87.3%)**	5 (7.0%)	0 (0%)	87.3
	Postmeno-pausal	0 (0%)	2 (3.5%)	**54 (94.7%)**	1 (1.8%)	94.7
	Male	1 (0.7%)	0 (0%)	0 (0%)	**139 (99.3%)**	99.3
					**Overall (%)**	94.5

Actual class (validation cohort; % classified)	(B)	*Classifier constructed using all data*				Validation accuracy (%)
		Predicted class (validation cohort)				
		OC user	Menstrual cycle	Postmeno-pausal	Male	
	OC user	**220 (83.7%)**	32 (12.2%)	7 (2.7%)	4 (1.5%)	83.7
	Menstrual cycle	20 (5.6%)	**318 (88.3%)**	14 (3.9%)	8 (2.2%)	88.3
	Postmeno-pausal	1 (0.4%)	16 (6.1%)	**241 (92.3%)**	3 (1.1%)	92.3
	Male	0	1 (0.2%)	0	**444 (99.8%)**	99.8
					**Overall (%)**	92.0

Actual class (validation cohort; % classified)	(C)	*Classifier constructed using ten most important variables*				Validation accuracy (%)
		Predicted class (validation cohort)				
		OC user	Menstrual cycle	Postmeno-pausal	Male	
	OC user	**215 (81.7%)**	40 (15.2%)	7 (2.7%)	1 (0.4%)	81.7
	Menstrual cycle	21 (5.8%)	**327 (90.8%)**	9 (2.5%)	3 (0.8%)	90.8
	Postmeno-pausal	2 (0.8%)	23 (8.8%)	**234 (89.7%)**	2 (0.8%)	89.7
	Male	0 (0%)	6 (1.3%)	0 (0%)	**439 (98.7%)**	98.7
					**Overall (%)**	91.4
